# Broadening the inherited *ASXL3* spectrum and unveiling molecular mechanisms through detailed genotypic-phenotypic analyses

**DOI:** 10.1016/j.gimo.2026.104409

**Published:** 2026-05-28

**Authors:** Emily Woods, Nicola Holmes, Catherine Gooch, Anne-Sophie Denommé-Pichon, Antonio Vitobello, Marie Vincent, Efterpi Pavlidou, Athina Ververi, Virginie J.M. Verhoeven, Marjon A. van Slegtenhorst, Abhijit Dixit, Ajoy Sarkar, Meena Balasubramanian

**Affiliations:** 1 Sheffield Clinical Genetics Service, Sheffield Children’s Hospital, Sheffield, United Kingdom; 2 Division of Clinical Medicine, School of Medicine and Population Health, University of Sheffield, Sheffield, United Kingdom; 3 Sheffield Diagnostic Genetics Service, Sheffield Children’s Hospital, Sheffield, United Kingdom; 4 Faculty of Biology, Medicine and Health, The University of Manchester, Manchester, United Kingdom; 5 Washington University, St Louis, WA; 6 Université Bourgogne Europe, CHU Dijon Bourgogne, Laboratoire de Génomique Médicale, FHU-TRANSLAD, Centre de recherche Translationnelle en Médecine moléculaire – Inserm UMR1231 équipe GAD, Dijon, France; 7 Centre Hospitalier Regional Universitaire De Dijon, Dijon, France/Service de Génétique Médicale, CHU de Nantes, Nantes, France; 8 Department of Paediatric Speech and Language Therapy, University of Ionnina, Ionnina Greece; 9 Department of Genetics for Rare Diseases, Papageorgiou General Hospital, Thessaloniki, Greece; 10 Department of Clinical Genetics, Erasmus University Medical Centre, Rotterdam, The Netherlands; 11 Nottingham Clinical Genetics Service, Nottingham City Hospital, Nottingham, United Kingdom; 12 The Bateson Centre, University of Sheffield, Sheffield, United Kingdom

**Keywords:** ASXL3, Bainbridge-Ropers, Genotype-phenotype, Mechanisms, Molecular

## Abstract

**Purpose:**

*ASXL3*-related disorder is a highly heterogeneous neurodevelopmental disorder, with increasing reports of inherited and recurrent variants indicating substantial intrafamilial phenotypic variability, as well as the possibility of interfamilial phenotypic variability.

**Methods:**

In this study, we conducted a detailed genotype-phenotype review of 204 individuals from the literature, including the International ASXL3 Natural History Study (IRAS: 316055). Statistical comparisons were made between individuals with variants leading to no protein product (nonsense-mediated messenger RNA decay [NMD], *n* = 87) and those with protein-truncating variants (no-NMD, *n* = 117). Phenotypes in 2 mutational clusters, mutational cluster region 1 (MCR1) (c.1095_2237, exon 11; *n* = 66) and mutational cluster region 2 (MCR2) (c.3043_4906, exon 12; *n* = 101), were also analyzed. Clinical details of individuals with recurrent variants were compared, along with a visual representation of the genotypes associated with some rarer clinical presentations. We also describe the clinical details of families with inherited *ASXL3* variants.

**Results:**

Microcephaly, sleep apnea, hyperventilation, and feeding tube use had a statistically increased prevalence in the NMD and MCR1 groups. Intellectual disability and global developmental delay were more severe in the NMD and MCR1 groups and were significant for the MCR1/MCR2 comparison (*P* = .0031 and *P* = .0183). Although autistic features were observed across all groups, the no-NMD and MCR2 cohorts had a higher proportion of individuals with formal autism diagnoses.

**Conclusion:**

We describe extensive phenotypic variability through the largest-to-date series of families with inherited pathogenic or likely pathogenic *ASXL3* variants (as per the American College of Medical Genetics and Genomics classification) and a review of the clinical details of unrelated individuals with recurrent variants. The family reports emphasize the possibility of additional, yet currently unidentified, factors influencing phenotypic expression or, alternatively, may reflect uncertainty about the pathogenicity of inherited *ASXL3* variants. Our data set facilitates comprehensive molecular comparisons to elucidate genotype-phenotype correlations, overcoming the limitations of previous studies with small sample sizes or an absence of statistical analysis. These findings contribute to a deeper understanding of the underlying pathophysiological mechanisms.

## Introduction

The *ASXL3* gene (OMIM: 615115; HGNC: 29357), identified in 2004, is the third member of the additional sex combs-like (ASXL) gene family.^1^ This family encodes putative polycomb proteins that are involved in transcriptional regulation and are thought to have an important role in neurodevelopment. Although the exact function of the ASXL3 protein has not been fully elucidated, it is believed to participate in the formation of a protein complex that functions as a histone methyltransferase, a crucial activity for chromatin remodeling.^2^

The *ASXL3* gene codes for the ASXL3 protein, which has 5 conserved domains: the ASXL N-terminal domain coded by exons 1 to 4 (codon 1-84), the ASX homology domain coded by exons 8 to 11 (codon 249-363), the ASXL motif 1 and 2 domains (ie, ASXM1 and ASXM2) coded by exon 11 (codon 963-1058) and exons 11 to 12 (codon 1741-1765), respectively, and the plant homeodomain coded by exon 12 (codon 2198-2248).^1^

*ASXL3*-related disorder (MIM: 615485) is a neurodevelopmental condition commonly associated with global developmental delay (GDD) or intellectual disability (ID), with varying degrees of behavioral issues, feeding and growth difficulties, and musculoskeletal abnormalities, among other features.^3^ This rare genetic disorder arises from loss-of-function variants in *ASXL3*, located on chromosome 18q12.1, and was first identified as a distinct disorder by Bainbridge et al^4^ in 2013. Since then, the disorder has shown substantial phenotypic variability.^5^

**Figure 1:**
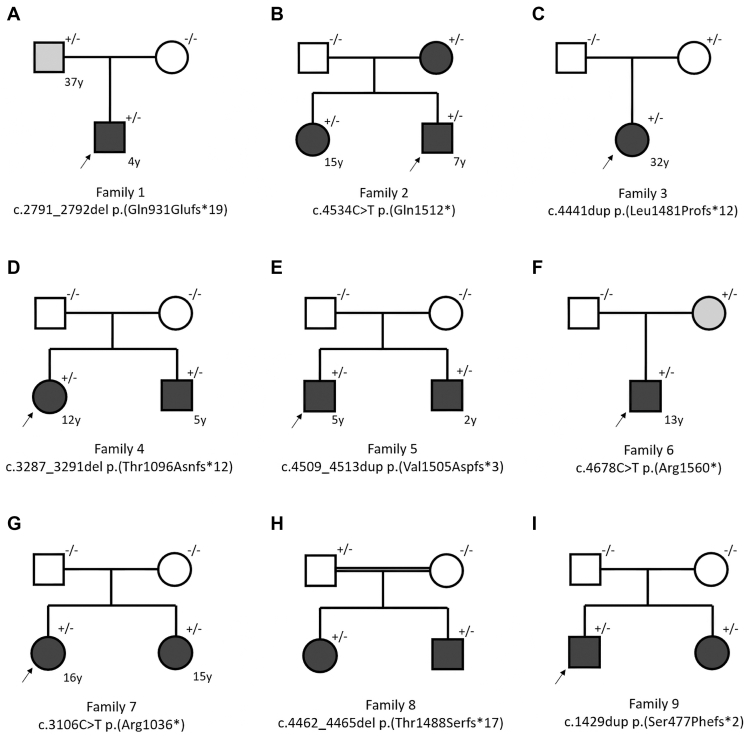
**Pedigreesof families in the existing literature with inherited *ASXL3*.** Black squares/circles represent individuals with a clinical diagnosis of *ASXL3*-related disorder. Gray squares/circles represent individuals with a milder phenotype, or symptoms which may be related to *ASXL3*. Individuals heterozygous for the family variant are depicted with the symbol +/-, whereas those individuals without the variant are depicted with the symbol -/-. Included ages were reported at the time of consultation in the literature. Families 1, 2 and 3 were described by Schirwani et al,^7^ families 4 and 5 were described by Schirwani et al,^6^ family 6 was described by Nagy et al,^8^ family 7 was described by Koboldt et al,^9^ family 8 was described by Bartolomaeus et al,^10^ and family 9 was described by Pande et al.^11^

Limited cases involving inherited *ASXL3* variants have been reported, demonstrating reduced penetrance and variable expressivity (Figure 1).^6^, ^7^, ^8^, ^9^, ^10^, ^11^ Although germline variants were confirmed in some instances, there are also reports of nonidentical siblings with the same apparent de novo variant, consistent with parental gonadal mosaicism (Figure 1D, E, G, and I).

The majority of individuals with *ASXL3*-related disorder have de novo variants in the final 2 exons, namely exons 11 and 12, with variant clustering in the first half of each exon.^12^ Variation resulting in a premature termination codon can trigger degradation of the messenger RNA (mRNA) transcript through nonsense-mediated mRNA decay (NMD), which is a protective surveillance pathway that reduces the production of potentially harmful truncated proteins.^13^ Variants causing a premature termination codon in exons 1 to 11 of *ASXL3* (except the final 50-55 nucleotides of exon 11) are expected to trigger NMD, whereas those in exon 12 would not.

Early studies proposed that truncating variants in *ASXL3* could have a dominant negative effect based on their presence in the penultimate exon, which might separate the protein's scaffolding function from its chromatin-targeting role.^4^^,^^14^ Subsequent research supported haploinsufficiency as the likely disease mechanism, because fibroblast studies did not produce a stable protein, with ASXL3 expression reduced by 50% compared with controls.^2^

A Xenopus model showed that a truncated ASXL3 protein could still rescue neural marker expression despite missing key functional domains, suggesting contrary to a dominant negative effect.^15^ This prompted the hypothesis that ASXL3 function might be reduced by NMD or lower protein levels, with truncated proteins potentially retaining partial functionality.^15^ If haploinsufficiency is the primary mechanism, variants that induce mRNA degradation may cause more severe phenotypes compared with those that generate truncated proteins, assuming no toxic gain-of-function.

Bainbridge et al^4^ proposed that *ASXL3* variants located at the 5′ end may result in a more severe phenotype compared with those at the 3′ end, based on the presence of 3′ variants in unaffected controls. Yu et al^16^ subsequently performed a limited genotype-phenotype analysis comparing individuals in the 5′ mutational cluster region (MCR) of exon 11 with the 3′ MCR of exon 12 and found no significant differences except for a higher incidence of hypertelorism in the 3′ MCR group. This analysis was limited by the numbers.

A more recent analysis of a Spanish cohort compared the clinical features of individuals with 3′ MCR variants to those with 5′ MCR variants and found that individuals with variants in the 3′ MCR showed more perinatal feeding issues, whereas those with variants in the 5′ MCR had lower height and occipitofrontal circumference (OFC) percentiles.^17^ These findings were limited to a cohort of 22 individuals and lacked power or statistical analysis.

Here, in this study, we perform a comprehensive genotype-phenotype analysis, comparing 5′ with 3′ MCR variants, as well as individuals with variants predicted to result in either no protein product (ie, NMD) or truncated proteins (ie, no-NMD).

## Materials and Methods

### Cohort selection

**Figure 2:**
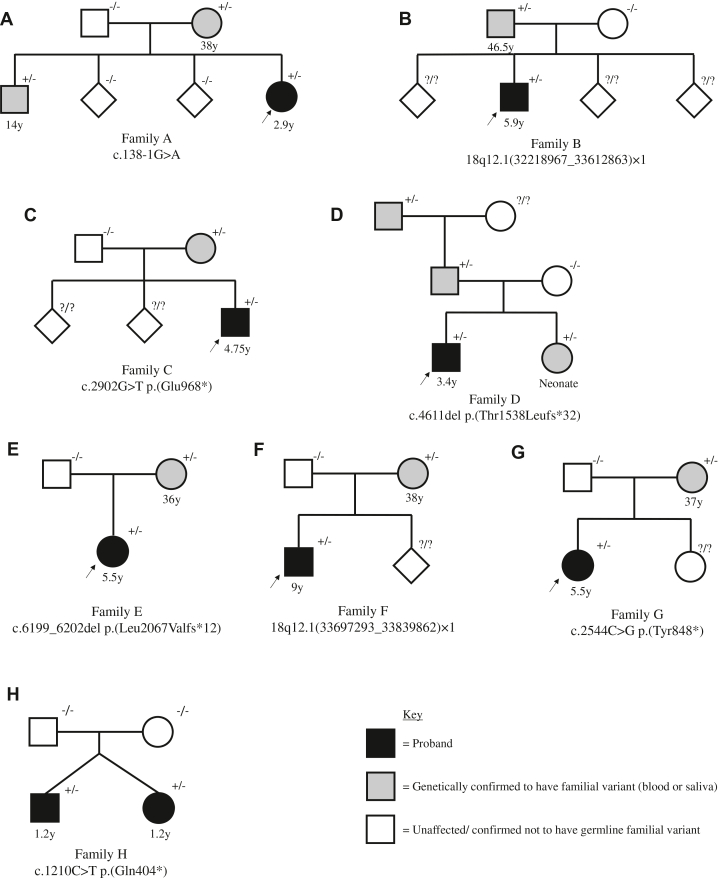
**Pedigrees of newly described families with inherited *ASXL3*.** Black squares/circles represent individuals with a clinical diagnosis of *ASXL3*-related disorder. Gray squares/circles represent individuals with a milder phenotype, or symptoms which may be related to *ASXL3*. Individuals heterozygous for the family variant are depicted with the symbol +/-, while those individual without the variant are depicted with the symbol -/-. Included ages were reported at the time of consultation or contact.

The following familial case series includes families from the ASXL3 Natural History Study (IRAS: 316055) (Figure 2: families A, B, E, F, G, and H) and cases identified through clinician communication (Figure 2: families C and D) with consent for publication. For molecular comparison, individuals were sourced from the literature, including the ASXL3 Natural History Study,^5^ and through direct clinician communication. An initial cohort of 103 individuals from Woods et al^12^ was cross-referenced with the ASXL3 gene page on the Human Genetic Mutation Database, yielding 352 references and 231 variants (searched on February 15, 2024). Duplicates, controls, corrigenda, and individuals with non-specific phenotypes or alternative diagnoses were excluded. A Google Scholar and PubMed search (on June 19, 2024) using ASXL3 and Bainbridge-Ropers identified 7 additional individuals not listed in Human Genetic Mutation Database, resulting in a final cohort of 204 individuals.

### Variant analysis

Variants were classified according to the relevant American College of Medical Genetics and Genomics criteria^18^, ^19^, ^20^ and the UK Association for Clinical Genomic Science best practice guidelines.^21^^,^^1^ All variants are described using the transcript reference NM_030632.3. Genomic coordinates are described using the GRCh38 reference genome (NC_000018.10).

### Phenotypic analysis

Deep phenotypic data were collected using a standardized proforma. Missing or unassessed data were excluded from the analysis. Where clinical details were otherwise extensive, omission was implied as indicating feature absence. Growth parameters were normalized using Z-scores from World Health Organization growth charts.^25^ Dysmorphic features were excluded due to the subjectivity of assessment, except for microcephaly (defined as an OFC below the third percentile). ID severity was scored from 0 (none) to 4 (profound) based on the described or quantified severity in the respective literature. GDD severity was similarly scored, with a maximum score of 3 (severe).

### Genotypic analysis

Variants leading to no protein product (via NMD, non-stop decay, or large deletions) were grouped as NMD (*n* = 87) and compared with the no-NMD group (*n* = 117), which comprised variants predicted to result in protein truncation. Two mutational clusters, MCR1 (NM_030632.3:c.1095_2237, exon 11, g.33738499_33739641) (*n* = 66) and MCR2 (NM_030632.3:c.3043_4906, exon 12, g.33742891_33744754) (*n* = 101), were defined based on regions with fewer variants. These cut-offs approximately correspond to the first half of each exon and are flanked by regions with a notably lower density of variants. Despite considerable overlap between the NMD and MCR1 groups and the no-NMD and MCR2 groups, analysis of both allowed a more distinct comparison. Any discrepancy between MCR1/MCR2 and NMD/no-NMD may be due to the inclusion of less established variant types, such as copy number variants and splicing variants, in the NMD/no-NMD analysis.

### Statistical tests

Comparisons of binary data between the groups were assessed for significance using a two-tailed Fisher’s exact test. A two-tailed *t-*test was applied to compare numerical data, including growth parameters, whereas a Mann-Whitney *U* test was used to analyze the severity of ID/GDD. The observed frequency distribution of variants along the length of the *ASXL3* gene in both controls and affected individuals was compared to the expected distribution using a Pearson’s χ^2^ test. A χ^2^ calculator was used to determine the *P* value.

## Results

### Family case summaries

#### Family A

Family A (Figure 2A) comprises a 2.9-year-old female proband, her 14-year-old brother, and her 38-year-old mother. The intron 2 c.138-1G>A splice variant (g.33644893G>A) was first identified on exome sequencing in the proband following investigations into developmental delay and autism.

The proband was born at 36 weeks via elective caesarean section because of poor growth, weighing 2.55 kg (5.5th centile). She presented with hypotonia (primarily in the upper body) and plagiocephaly, which was treated with helmet therapy. Her development was globally delayed. She preferred independent and repetitive play and maintained physical boundaries. She was diagnosed with autism spectrum disorder (ASD) at 18 months.

Feeding difficulties included lip/tongue-tie and reflux. She had a prolonged tongue thrust when eating solids, requiring small pieces of food. Now, she is generally calm, enjoys water, and occasionally exhibits inappropriate laughter. Recent growth centiles are weight 70th, height 86th, and head circumference 63rd.

**Figure 3:**
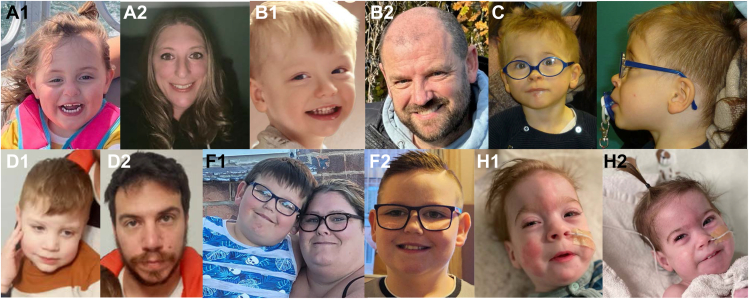
**Facial photographs of individuals described in the family case summaries**. A1. Proband of family A with facial features include prominent eyebrows, sunken eyes, and broad nasal tip. Additional physical features include high arched palate, overbite, shorter neck, and inverted nipples. A2. Mother of family A with wide nasal bridge, broad nasal tip, low columella, high, narrow palate, and overbite, previously requiring Orthodontic treatment for dental overcrowding. B1. Proband of family B with thin upper lip, wide nasal bridge, and nasal tip. B2. Father of family B with no overt dysmorphology. C. Front and side profile of proband of family C with prominent forehead, broad nasal tip, large mouth, high palate, and frequent drooling. D1. Proband of family D with trigonocephaly, prominent forehead, down-slanting eyes, epicanthic folds, and broad nasal tip. Additional features include sparse teeth and brittle nails. D2. Father of proband family D, with similar facial features to his son including prominent forehead, down-slanting eyes, epicanthic folds, broad nasal tip, and long face. F1. Mother and son from family F with similar facial features. F2. Proband of family F with epicanthic folds, sparse eyebrows, almond-shaped eyes, left earlobe crease, and thin lips. H1. Male twin of family H with prominent forehead, down-slanting palpebral fissures, epicanthic folds, prominent eyebrows, long eyelashes, wide nasal bridge, wide nasal tip, small mouth and chin, metopic ridge, and high arched palate. In addition, he has positional talipes, positional wrist and thumb contractures, and mild pectus excavatum. H2. Female twin from family H with down-slanting palpebral fissures, synophrys, long eyelashes, wide nasal bridge, small mouth and chin, high palate, and pointed ears. In addition, she has positional talipes, a milder positional wrist flexion and pectus excavatum.

The mother had typical development. She was diagnosed with epilepsy at age 8, with 2 seizures annually until age 14, but has remained seizure-free without medication since then. She developed myopia at age 18 and still wears glasses. She completed higher education and works as a paralegal. She shares some physical features with her daughter (Figure 3A1 and A2). Recent growth centiles are weight 63rd and height 31st.

The brother was born via emergency caesarean section at 36 weeks, weighing 3.32 kg (17th centile). An amniocentesis was performed because of a high trisomy risk, but no abnormality was detected on the microarray. He required ventilation for 5 weeks and was treated for presumed chorioamnionitis, but achieved normal developmental milestones. Early autistic traits (eg, lining up toys and solitary play) resolved; he is now sociable, is achieving academically, and is occasionally hyperactive. Recent growth centiles are weight 78th and height 18th.

#### Family B

Family B (Figure 2B) comprises a 5.9-year-old proband and his 46.5-year-old father, with a heterozygous 1.4 Mb interstitial loss of the long arm of chromosome 18 within band q12.1 (18q12.1(32218967_33612863) × 1, GRCh38), confirmed by microarray. The deletion includes 4 protein-coding genes (non-OMIM-morbid) plus exons 1 to 2 of *ASXL3*. This deletion was identified in the proband during the investigation of delayed development and learning.

The proband was born at term following an uncomplicated pregnancy, weighing 3.54 kg (49th centile). He first sat independently at 12 months and walked independently at 18 months. He experiences fatigue after approximately 10 minutes of walking and still falls frequently. Fine motor coordination was delayed; he can hold a pencil in a fist-grasp but can write his name. His first words were at 2 years, and sentence formation at 5 years. Articulation remains poor, though receptive language exceeds expressive language. He previously used communication cards but now gestures to express his needs. He now has occasional behavioral meltdowns, engages in solitary play, and exhibits stereotypical behaviors, awaiting assessment for ASD. He has mild hypotonia, hypermobility, intermittent toe-walking, and mild strabismus.

Feeding history included breast and bottle feeding until 6 months, though the diet now remains limited by sensory issues. Current educational support includes 1:1 assistance and psychological input because of school refusal. Recent growth centiles are weight 82nd and height 26th.

The proband’s father recalled delayed development, poor school performance, distractibility, and social challenges. He is awaiting assessment for ASD and attention deficit hyperactivity disorder (ADHD). His medical history includes hypertension, hypothyroidism, high cholesterol, and anxiety with adult-onset obsessive-compulsive behaviors. He has worked in various jobs but is now retired on medical grounds and manages childcare duties. Recent growth centiles are height 31st and weight 84th.

#### Family C

A 4.75-year-old male proband and his mother carry a c.2902G>T p.(Glu968Ter) *ASXL3* variant (g.33740306G>T), located in exon 11 (outside of MCR1 group). This variant was identified on genome sequencing in the proband at age 3 (Figure 2C). In addition, the proband harbors a de novo 2.23 Mb interstitial loss at 17q23.

The pregnancy was complicated by intrauterine growth restriction and pyelocaliceal dilatation. He was born by vaginal delivery at 36 weeks, weighing 1.765 kg (first centile), with a head circumference of 30.2 cm (third centile).

Gross motor milestones were achieved on time, whereas fine motor skills and speech development were delayed. He remains nonverbal but uses signs to communicate. He has strabismus, nystagmus, and astigmatism, requiring prescription glasses. Additional diagnoses include asthmatic bronchitis and testicular ectopy.

Feeding difficulties and gastroesophageal reflux were apparent in the neonatal period but resolved at approximately 1 year. Now, his behavior can be restless, with poor sleep, for which he is taking melatonin. He currently requires additional educational support. Recent growth centiles are weight seventh, height third, and head circumference third.

His mother had typical early development. She obtained a professional diploma and now works as a personal care assistant. Her medical history includes mild gastrointestinal issues (dolichocolon and reflux) and mild anxiety. Her growth centiles are height 31st and head circumference 98th.

#### Family D

A paternally inherited c.4611del p.(Thr1538LeufsTer32) variant (g.33744459del), located on exon 12 and within MCR2, was first identified by genome sequencing in the male proband at age 3.4 years. Testing confirmed its inheritance across three generations (Figure 2D).

The proband was born at term, weighing 3.95 kg (80th centile) with a head circumference of 36 cm (83rd centile). He sat independently at 7 months and walked at 2 years and 3 months. His first words were spoken at 12 months, but he remains nonverbal. He had central hypotonia, poor visual-motor coordination, and surgically corrected trigonocephaly. At 2.5 years, he developed strabismus, and renal imaging showed a dysplastic kidney but without renal impairment. Recent growth centiles are height 91st, weight 93rd, and head circumference 85th.

The proband’s father had a speech delay, with his first words at 5 years, and still has residual articulation difficulties. He experienced significant learning difficulties as a child and likely has mild ID (though not formally assessed). He works in a supermarket warehouse. The paternal grandfather also had a speech delay, unclear articulation, and faced mild cognitive difficulties. He worked as a farmer after limited schooling.

The proband’s female sibling (neonate) inherited the same variant and has mild generalized hypotonia, reduced head and neck control, microcephaly, and overlapping metopic sutures; further investigations are ongoing.

#### Family E

Family E consists of a 5.5-year-old female proband and her 36-year-old mother, who has a c.6199_6202del, p.(Leu2067ValfsTer12) variant (g.33746047_33746050del) identified on trio exome sequencing (Figure 2E). This variant is located on exon 12 but is outside MCR2.

The pregnancy was complicated by growth restriction, resulting in an emergency caesarean section at term (birth weight 2.4 kg, first centile). The proband’s early motor development was typical, but she is now relatively uncoordinated in her movements. She was diagnosed with ASD at age 4, her language development was delayed, and she remains mainly nonverbal, communicating with gestures and devices. Early feeding difficulties included reflux, and she struggled with the transition to solids, but now eats well. She has hypermobility of some joints and a history of upper respiratory infections, which improved after adenotonsillectomy.

She is described as happy but stubborn, with a short attention span, and she tends to observe play. She attends a special nursery for children with developmental delays. Facial features include a prominent forehead, subtle hypertelorism, bushy eyebrows, a prominent philtrum, and widely spaced teeth. Additional features include a single palmar crease on one hand and blocky fingers. Recent growth centiles are weight 34th and height 68th.

Her mother’s early development was typical, though she was diagnosed with ADHD. She has hypermobility, requiring physiotherapy, and experiences pelvic instability and gynecological issues. She has a prominent forehead, down-slanting palpebral fissures, a wide nasal bridge, and a broad nasal tip. Her height is at the 31st centile.

#### Family F

Family F comprises a 9-year-old male proband and his 38-year-old mother, who have a 143 kB 18q12.1(33697293_33839862) × 1 deletion (GRCh38) identified on microarray (Figure 2F). This is an *ASXL3* multi-exon deletion (exons 9-12).

The pregnancy was uneventful, except for the use of low molecular weight heparin for venous thromboembolism risk. The proband was born at 37 weeks by elective caesarean section, weighing 3.26 kg (26th centile). Development was as expected until age 4, when social and behavioral concerns arose. He was diagnosed with ADHD at age 8, still takes methylphenidate, and struggles with social interactions and impulsivity, including self-injury and emotional dysregulation. He is behind with learning, but he has no speech and language or motor delay. He has had frequent respiratory infections in the past and borderline sleep apnea. He also has mild strabismus and wears glasses for myopia.

He attends a mainstream school with support. He has never had any issues with poor feeding or growth; he eats well, has a good appetite, and is now overweight (Figure 3F1 and F2). Recent growth centiles are weight >99th and height 77th.

The proband’s mother was behind in her early development compared with her siblings. As a child, she attended a special primary school and left school before achieving any qualifications. She had strabismus corrected surgically and now wears glasses for hypermetropia. She has had one tonic-clonic seizure. She has obstructive sleep apnea and is on continuous positive airway pressure (CPAP) at night, as well as experiencing migraines, anxiety, and depression. She struggles with emotional regulation. She is a full-time caregiver for her son.

#### Family G

A c.2544C>G p.(Tyr848Ter) variant (g.33739948C>G), located on exon 11, was incidentally identified by trio genome sequencing in the 5.5-year-old proband during an acute encephalopathic episode and was found to be inherited from her 37-year-old mother (Figure 2G).

The female proband, who is also described by Woods et al,^5^ was born at term with no complications, weighing 3.69 kg (74th centile), and achieved all developmental milestones. There were no issues with social interactions, feeding, or growth. At age 4, she was hospitalized for sudden-onset lethargy, confusion, and unresponsiveness, requiring brief intubation. Despite extensive testing, the cause of the encephalitis remained unknown. She recovered over several weeks, and follow-up investigations were normal. After the illness, she struggled with emotions, leading to temper tantrums, but she has no self-injurious behaviors. She attends a mainstream school with no learning concerns. There are no dysmorphic features. Recent growth centiles are weight 63rd and height 33rd.

The mother had no neonatal or developmental issues. She has no medical issues but wears glasses for myopia and astigmatism. She has completed higher education in Health and Social Care. Her recent growth centiles are weight 76th and height 46th.

#### Family H

Both 1.2-year-old non-identical twins (male and female) were diagnosed with the recurrent, apparent de novo, c.1210C>T p.(Gln404Ter) variant (g.33738614C>T), likely as a result of parental gonadal mosaicism (Figure 2H). This variant is located on exon 11 of MCR1. Exome sequencing was first initiated in the male sibling at 3 months. Targeted testing of the familial variant later confirmed that it was also present in the female sibling.

The twins were born preterm at 33+1 weeks via emergency caesarean section because of pre-eclampsia, with corrected birth weights of 2.06 kg (52nd centile) and 1.72 kg (26th centile), respectively. Both siblings were admitted to the neonatal intensive care unit for breathing and feeding support, with the male twin requiring CPAP for 4 days. Bronchoscopy confirmed tracheomalacia, and a cranial ultrasound scan revealed corpus callosum agenesis. He had constipation and feeding difficulties with persistent vomiting, leading to the insertion of a percutaneous jejunostomy tube at 13 months because of slow weight gain. Generalized hypotonia and repetitive head movements were noted, with a normal electroencephalography. At night, he sometimes uses a humidifier with an integrated flow generator machine (Airvo) for occasional respiratory distress.

The female twin also required a brief time on CPAP after birth and subsequent nasogastric feeding. She had left lateral ventriculomegaly and a cramped foramen magnum. Her development and feeding patterns were similar to her brother's. She exhibited repetitive head movements and body tensing, with a normal electroencephalography. Her sleep was more disturbed.

Growth centiles for both siblings were: less than third centile for weight and height, whereas the male twin had relative macrocephaly on the 27th centile.

### Molecular comparisons

**Figure 4:**
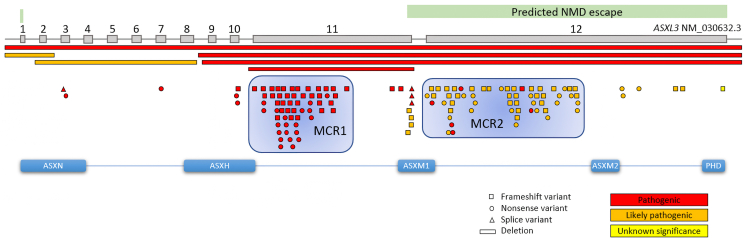
**Location of variants identified in individuals with *ASXL3*-related disorder that were included for analysis.** The type of variant is represented by the type of shape, and the American College of Medical Genetics and Genomics classification is represented by the color of the shape. Variants that fall within the predefined clusters, mutational cluster region 1 (MCR1) and mutational cluster region 2 (MCR2), are highlighted. MCR, mutational cluster region.

Of the 204 individuals, 105 were male, 89 were female, and the remaining were unspecified. Ages ranged from prenatal to 47 years (median age: 6 years). There were 76 pathogenic variants, 62 likely pathogenic variants, and one variant of uncertain significance (VUS) (Figure 4). The VUS, which was the most 3′ variant reported, was included because it was regarded by Verhoeven et al^26^ as the likely cause of the individual’s phenotype. There were 139 different variants, comprising 54 nonsense variants, 75 small insertions/deletions, 4 splicing variants, and six gross deletions, as summarized in the Supplemental Material 1.

A Pearson χ^2^ test (Supplemental Material 2) assessed whether variants in this cohort were overrepresented in exons 11 and 12 relative to their sizes. The test showed a significant result for the entire gene (χ^2^_11_ = 31.79, *P* = .000824), indicating an uneven variant distribution. Exon 11 had a higher-than-expected number of variants (χ^2^_1_ = 20.94, *P* = .00028), whereas while exon 12 did not (χ^2^_1_ = 1.95, *P* = .377).

A summary of phenotypic characteristics is provided in Supplemental Material 3. There were no statistically significant differences in antenatal and neonatal phenotypes between NMD/no-NMD groups or between MCR1/MCR2 groups. This included pregnancy complications, type of birth, gestation, admission to the neonatal intensive care unit, birth weight, and birth OFC.

**Figure 5:**
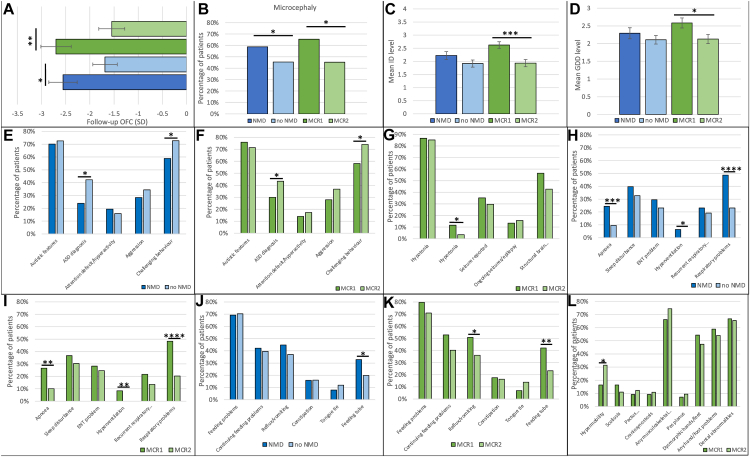
**Statistically significant findings on comparison between nonsense-mediated messenger RNA decay (*NMD*)/no nonsense-mediated mRNA decay (no-NMD) and mutational cluster region 1 (MCR1)/mutational cluster region 2 (MCR2) groups.** A. Comparison of occipitofrontal circumference (OFC), as per latest measurement. Mean OFC based on Z score for each individual, normalized for sex and age. B. Microcephaly recorded where stated, or if OFC was less than third centile. C. Mean intellectual disability (ID) level by group, where 0 = no ID, 1 = mild, 2 = moderate, 3 = severe, and 4 = profound. D. Mean global developmental delay (GDD) level by group, where 0 = no GDD, 1 = mild, 2 = moderate, 3 = severe, and 4 = profound. E. Comparison of the social and behavioral phenotypes between the NMD/no-NMD groups. F. Comparison of the social and behavioral phenotypes between the MCR1/MCR2 groups. G. Comparison of neurological findings between MCR1/MCR2 groups. H. Comparison of the sleep and respiratory phenotypes between the NMD/no-NMD. Ear, nose, and throat problems include enlarged tonsils, otitis media, and choanal atresia. “Respiratory problems” is collated data from apnea, hyperventilation, recurrent respiratory infections, and miscellaneous respiratory problems. I. Comparison of the sleep and respiratory phenotypes between the MCR1/MCR2. ENT problems include enlarged tonsils, otitis media and choanal atresia. “Respiratory problems” is collated data from apnea, hyperventilation, recurrent respiratory infections and miscellaneous respiratory problems. J. Comparison of the feeding and gastrointestinal phenotypes between the NMD/no-NMD groups. Feeding problems include difficulty latching as a baby, poor appetite, and any individual reported to have feeding difficulties, which may be inclusive of reflux/vomiting. Feeding tube included nasogastric tube, percutaneous endoscopic gastrostomy tube, and jejunostomy tube. K. Comparison of the feeding and gastrointestinal phenotypes between the MCR1/MCR2 group. Feeding problems include difficulty latching as a baby, poor appetite, and any individual reported to have feeding difficulties, which may be inclusive of reflux/vomiting. Feeding tube included nasogastric tube, percutaneous endoscopic gastrostomy tube, and jejunostomy tube. L. Comparison of the musculoskeletal phenotypes between the MCR1/MCR2 groups. Error bars represent the standard error of the mean.∗*P* < .05, ∗∗*P* < .01, ∗∗∗*P* < .005, ∗∗∗∗*P* < .001. ASD, autism spectrum disorder; NMD, nonsense-mediated messenger RNA decay; MCR, mutational cluster region.

There was no statistically significant difference between the follow-up values for height and weight among the groups. However, follow-up OFC was significantly smaller in the NMD and MCR1 groups than in the no-NMD and MCR2 groups (Figure 5A and B). Although representing a smaller cohort, Trujillano et al^17^ also determined a smaller head size in their 5′ MCR cohort of 22 Spanish individuals (details of which were not included in our analysis).

There was no significant difference in the age at which major developmental milestones were met in each group (eg, independent sitting, independent walking, first smile, and first words), nor whether individuals had ongoing speech and language issues (including whether they were non-verbal). However, on average, ID and GDD were more severe in the NMD and MCR1 groups, and this was significant for the MCR1/MCR2 comparison with relevance to both ID and GDD (*P* = .0031 and *P* = .0183) (Figure 5C and D).

The proportion of individuals with autistic features was similar across cohorts, but the no-NMD and MCR2 groups significantly had more individuals with a formal ASD diagnosis than the NMD and MCR1 groups (Figure 5E). Various factors, including challenging behaviors (eg, aggression, self-injury, and breath-holding), which were more prevalent in the no-NMD and MCR2 groups (Figure 5F), may have influenced the pursuit of a formal ASD diagnosis. However, the severity of GDD/ID did not statistically correlate with the likelihood of an ASD diagnosis (*P* = .28). Although a formal ASD diagnosis and challenging behaviors were statistically increased in the MCR2 and no-NMD groups, there are many potential influencing factors to consider concerning behavioral and social phenotypes.

Hypotonia was observed in 80% to 90% of individuals across all groups. Although less common, a higher proportion of individuals in the NMD and MCR1 groups exhibited hypertonia, with the MCR1 group showing a significantly higher rate than the MCR2 group (Figure 5G).

There was no significant difference between the proportions of individuals reported to have strabismus, other vision problems, or hearing problems. Respiratory problems were relatively more common in the NMD and MCR1 groups (*P* = .002). When broken down by phenotype, there were significant differences in hyperventilation and apnea (Figure 5H and I).

There was no significant difference in the proportion of individuals with feeding problems, but the MCR1 group had significantly more individuals with a history of reflux and/or vomiting than the MCR2 group. The proportion of individuals who were ever fed via a feeding tube was significantly higher in the NMD and MCR1 groups than in the no-NMD and MCR2 groups (Figure 5J and K).

Hypermobility was significantly more common in the MCR2 group than in the MCR1 group. No significant differences were observed between the cohorts for other commonly reported musculoskeletal phenotypes (Figure 5L).

### Recurrent variant comparison

The most commonly recurring variants were c.3106C>T p.(Arg1036Ter) (g.33742954C>T) and c.4330C>T p.(Arg1444Ter) (g.33744178C>T), each of which was identified in 8 different individuals. The c.1210C>T p.(Gln404Ter) (g.33738614C>T) and c.4399C>T p.(Arg1467Ter) (g.33744247C>T) variants, each of which was identified in 5 different individuals, whereas c.3349C>T p.(Arg1117Ter) (g.33743197C>T) was identified in 4 unrelated individuals. All of these variants are located in CpG dinucleotides, which have an increased propensity for de novo variation, particularly C > T transitions.^27^

A phenotypic comparison of individuals with the same variant, seen in 4 or more cases (excluding proven inherited cases), is summarized in Supplemental Material 4. Because of insufficient sample sizes for recurrent variants, meaningful power calculations cannot be performed. However, stacked charts in Supplemental Material 4 visually depict common phenotypic differences.

### Rare phenotype comparison

**Figure 6:**
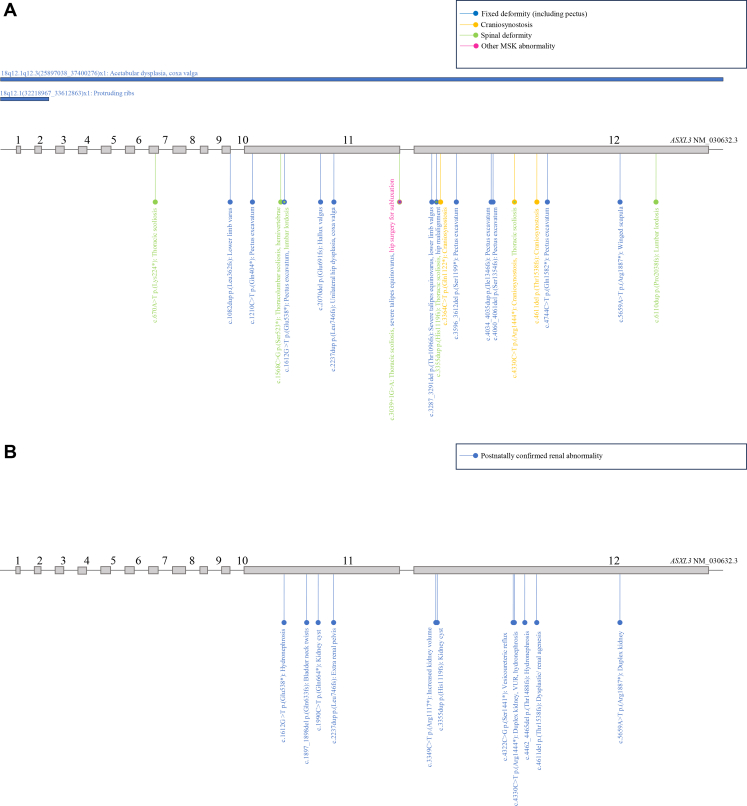
**Location of variants and description of rarer phenotypes.** A. Rarer musculoskeletal (MSK) phenotypes. B. Postnatally confirmed renal abnormalities.

To further investigate any potential genotypic correlation for rarer phenotypes, cases were identified, and then variants were plotted on a gene diagram to illustrate their location. This showed a variety of gene locations for rarer musculoskeletal phenotypes (Figure 6A) and postnatally confirmed anomalies of the kidney or urinary tract (Figure 6B). This further suggested that there is a lack of genotypic influence on rarer phenotypes.

## Discussion

### Genotype-phenotype correlation

This study hypothesized that if haploinsufficiency is the main disease mechanism, genetic variants causing degradation of the mRNA transcript through NMD would produce more severe clinical outcomes than variants that generate truncated proteins (assuming there may be residual function and no toxic gain of function). Variants were therefore grouped according to whether they were predicted to trigger NMD and, separately, by MCRs within exons 11 and 12 (MCR1 and MCR2).

Somewhat in support of the hypothesis, analyses revealed that individuals in the NMD and MCR1 groups showed statistically significantly higher rates of certain clinical features, including microcephaly, sleep apnea, hyperventilation, and use of feeding tubes. Additional significant findings include greater severity of ID, GDD, reflux or vomiting, and hypertonia in the MCR1 group, with similar trends observed in the NMD comparison group.

However, these differences were not consistent across all measures, and a more severe overall phenotype was not observed in the NMD and MCR1 groups. Many different clinical parameters were compared in this analysis that were not found to have a statistically significant difference between groups. This may reflect external influencing or predisposing factors or the multiple etiology of certain medical problems (such as seizures). This may also be due to the smaller sample size of certain features, for example, musculoskeletal features. Of note, assessments of ID/GDD severity were not standardized or conducted by a single evaluator, but were derived from reported descriptions in the literature, which may reflect subjective clinical judgment or varying assessment methods.

Contrary to our hypothesis, behavioral difficulties and diagnoses of ASD were reported more often in individuals in the no-NMD and MCR2 groups. This may partly reflect diagnostic bias; the journey to an autism diagnosis can be complex and may or may not be pursued in addition to a genetic diagnosis. However, it is noteworthy that no direct relationship was observed between autism diagnoses and the severity of cognitive impairment in this dataset. Furthermore, behavioral difficulties may represent a more subjective measure; therefore, comparisons involving such parameters, including sleep disturbance and aggression, should be interpreted with caution.

Any differences were generally greater when comparing MCR1 with MCR2 (compared with the NMD comparison), suggesting a clearer phenotypic distinction between these mutation clusters. This difference is likely because of the inclusion of less well-established variants in the NMD analysis, such as copy number variants (CNVs) and splice site variants.

Variants in exon 12 are not expected to trigger NMD but instead produce a truncated ASXL3 protein lacking the ASXM2 and the plant homeodomain that are involved in nuclear receptor interactions and chromatin regulation. Downstream variants may retain a functional ASXM2 domain. These domains are crucial for ASXL3 interaction with nuclear hormone receptors and histone modification recognition.^28^^,^^29^ Disruption could lead to transcriptional dysregulation, though whether truncated ASXL3 proteins are non-functional or retain some activity remains unclear. The pathogenicity of frameshift and nonsense *ASXL3* variants in exons 1 to 11 is often attributed to NMD, which prevents the translation of aberrant transcripts.

However, MacArthur et al^30^ found that only 25% of predicted NMD variants showed significant decay, with most having no impact on gene expression. NMD dynamics may also differ during the development.^4^ Alternatively, MCR1 variants may produce truncated ASXL3 proteins that are missing key domains, potentially disrupting transcription or rendering the protein non-functional. Lichtig et al^15^ showed that a truncated ASXL3 protein could partially rescue a phenotype in frog embryos, suggesting residual function.

Variant distribution analysis also suggested that loss-of-function mutations occur more frequently in exon 11 than expected, while exon 12 variants were less frequent in the gnomAD control cohort, implying that exon 11 may be less tolerant to disruption than exon 12. However, low-quality variant calls should be interpreted cautiously, especially in the 3′ region, where the most distal variant in *ASXL3*-related disorder occurs.^26^ These results suggest that exon 11 variants are more deleterious, though if NMD or larger deletions were responsible, variants in exons 1 to 10 would be similar or more harmful, which was not observed in control individuals. Predictions about NMD may not be reliable, as some variants expected to undergo decay may not show a significant reduction in gene expression.

Although there were some statistically significant differences between groups in our analyses, the biological interpretation of these results remains complex.

### Inherited variants

Given the substantial inter-familial variability observed in families with inherited *ASXL3* variants, it is plausible that additional genetic or non-genetic factors may influence the phenotypic expression. In some individuals, this variability may reflect a comparatively mild clinical presentation or suggest reduced penetrance.

For example, the non-specific neurodevelopmental phenotype in the proband of family E, and the possible mild/unrelated phenotype in the mother, could demonstrate the wide spectrum of the disorder. Alternatively, it could provide evidence for the benignity of this *ASXL3* variant. Only a small number of CNV deletions such as this have been reported to date, although haploinsufficiency is the expected pathogenic mechanism.

Similarly, although no alternative explanation was identified on exome sequencing in family A, the significant variability in this family is striking, leading to the question of whether this intronic *ASXL3* variant is truly disease-causing. Fewer intronic variants of this type have been reported to date, although future functional studies may help to clarify their effects.

Alternatively, these findings may indicate that certain inherited *ASXL3* variants actually exhibit limited pathogenic potential. The variant identified in family G was classified as a VUS by the reporting laboratory because of a lack of phenotypic fit; therefore, this family was not included in the genotype comparisons detailed in this study. However, this truncating variant would fit in the MCR1 region in exon 11. This case brings into question whether inherited *ASXL3* variants are disease-causing or not.

Notably, not all reported probands display phenotypes that are fully consistent with the initially characterized *ASXL3*-related disorder. Although within families there are shared familial facial features (as expected), overall dysmorphology is generally less pronounced among inherited cases than in cohorts of probands with a more typical presentation of *ASXL3*-related disorder (Figure 3).

There is also the consideration of potential dual diagnoses, as in family C. As 17q23 microdeletion syndrome presents with variable expressivity and incomplete penetrance, a possible composite phenotype cannot be excluded in this proband. However, as the mother has no clear features of *ASXL3*-related disorder, this family may represent a case of reduced penetrance or alternatively provide evidence of the possible benignity of this *ASXL3* variant.

### Limitations

The described families with inherited *ASXL3* variants show significant inter- and intra-familial variability. Accordingly, inherited variants should be interpreted with caution, and the possibility of an alternative diagnosis should be considered.

The proposed variant categories used for genotype–phenotype analyses were based on theoretical principles rather than functional evidence. Any observed discordance between MCR1/MCR2 and NMD/no-NMD groups may reflect the inclusion of less well-established variant types, such as CNVs. Similarly, the absence of functional validation necessitated reliance on the American College of Medical Genetics and Genomics classification alone. These inherited variants (with the exception of family G) were incorporated into the genotype analyses. Where there was a doubt regarding pathogenicity, this could have potentially affected the results.

Additional limitations stem from the nature of the available literature, including the young age of some individuals, which may have resulted in incomplete phenotypic characterization, as well as missing clinical data (which was addressed in the analysis). Moreover, heterogeneity in phenotyping methods across studies, rather than standardized evaluation by a single assessor, could have introduced variability.

### Conclusion

We expand on the vast variability of *ASXL3* variants, including a comparison of clinical features in unrelated individuals with recurrent variants and the largest-to-date detailed familial cases with inherited *ASXL3* variants, which include a described case of inherited *ASXL3* across three generations.

Our dataset explores molecular comparisons to identify genotype-phenotype correlations, addressing previous limitations of small sample sizes and a lack of statistical analysis. Microcephaly, sleep apnea, hyperventilation, and feeding tube use were more common in the NMD and MCR1 groups. Other significant findings, such as the severity of ID/GDD, reflux/vomiting, and hypertonia, were mostly seen in the MCR1/MCR2 comparison, with trends also observed in the NMD/no-NMD comparison.

This may be congruent with the mechanism of haploinsufficiency, with some residual activity of the truncated proteins. However, the phenotypic presentation is seemingly more complex than concluding that one variant type results in a more or less severe condition than another. However, it is not entirely clear whether this is entirely rooted in the pathogenesis of the disease or whether there are other influencing factors at play.

Despite observed statistical differences between variant cohorts, the clinical spectrum remains highly variable, even among unrelated individuals with the same variant. The spectrum described in the inherited *ASXL3* cases is striking, which may provide further evidence for external influencing factors or the possibility of reduced penetrance or provide evidence of the partial or complete benignity of inherited variants. Further functional studies are needed before any genotype-based clinical outcome predictions can be considered.

## Conflict of Interest

The authors declare no conflicts of interest.

## Footnotes

1 PVS1 was applied at very strong for all variants resulting in termination of translation before p.995, where nonsense-mediated messenger RNA decay is predicted to occur. This was downgraded to strong for those predicted to result in a truncated protein with more than 10% reduction in protein size (p.995_2022) and to moderate for termination at p.2023 onwards. PS2 was applied at a moderate level for de novo variants identified by trio exome sequencing/genome sequencing, since the phenotype is consistent but not highly specific to the *ASXL3* gene. Where de novo status was established by parental testing, but parentage was not confirmed by trio exome sequencing /genome sequencing, PM6 was applied at a supporting level. PP1 was applied for co-segregation in line with Jarvik and Browning.^22^ PM2 was applied only to variants not identified in any control participant in gnomAD v4.1.0.^23^ The assessment of splicing variants was based on predictions made by SpliceAI.^24^
