# Supplementary material for: Broadening the inherited ASXL3 spectrum and unveiling molecular mechanisms through detailed genotypic-phenotypic analyses
Source: Genet Med Open. 2026 May 28;4:104409. doi: 10.1016/j.gimo.2026.104409 (PMC13393785; doi:10.1016/j.gimo.2026.104409)
Supplement: Supplemental Material 1 [file mmc1.docx]

| **Reference first published** | **Patient** | **Variant** | **Genomic co-ordinates (GRCh38 NC_000018.10)** | **Consequence** | **Exon** | **Inheritance** | **ACMG criteria** | **Classification** | **NMD** | **MCR** |
| --- | --- | --- | --- | --- | --- | --- | --- | --- | --- | --- |
| Woods et al.^5^ | U34 / F10a (our Family A) | c.138-1G>A | g.33644893G>A | Likely skipping of exon 3 | Intron 2 | Maternal | PVS1, PM2 | Pathogenic | NMD |  |
| Woods et al.^5^ | U35 / F10b (our Family A) | c.138-1G>A | g.33644893G>A | Likely skipping of exon 3 | Intron 2 | Unknown | PVS1, PM2 | Pathogenic | NMD |  |
| Woods et al.^5^ | U36 / F10c (our Family A) | c.138-1G>A | g.33644893G>A | Likely skipping of exon 3 | Intron 2 | Maternal | PVS1, PM2 | Pathogenic | NMD |  |
| Schirwani et al.^3^ | L45 | c.187C>T | g.33644943C>T | p.(Arg63Ter) | 3 | *De novo* | PVS1, PS2_mod | Pathogenic | NMD |  |
| Woods et al.^5^ | U7 | c.670A>T | g.33671821A>T | p.(Lys224Ter) | 7 | Unknown | PVS1, PM2 | Pathogenic | NMD |  |
| Wang et al.^31^ | L58 | c.1063G>T | g.33734396G>T | p.(Glu355Ter) | 10 | *De novo* | PVS1, PM2, PM6_sup | Pathogenic | NMD |  |
| Balasubramanian et al.^32^ | L48 | c.1074T>A | g.33734407T>A | p.(Tyr358Ter) | 10 | *De novo* | PVS1, PM2, PS2_mod | Pathogenic | NMD |  |
| Balasubramanian et al.^32^ | L52 | c.1082dup | g.33734415dup | p.(Leu362AlafsTer23) | 10 | *De novo* | PVS1, PM2, PS2_mod, PS4_sup | Pathogenic | NMD |  |
| Woods et al.^5^ | U5 | c.1082dup | g.33734415dup | p.(Leu362AlafsTer23) | 10 | *De novo* | PVS1, PM2, PS2_mod, PS4_sup | Pathogenic | NMD |  |
| Lee et al.^33^ | L147 | c.1082+1784_3039+952del | g.33736199_33741395del | p.(Leu362PhefsTer11) | 11 | *De novo* | PVS1, PM6_sup, PS3_sup | Pathogenic | NMD |  |
| Schirwani et al.^3^ | L13 | c.1095_1096del | g.33738499_33738500del | p.(Glu367GlyfsTer17) | 11 | *De novo* | PVS1, PM2, PM6_sup | Pathogenic | NMD |  |
| Balasubramanian et al.^32^ | L47 | c.1201del | g.33738605del | p.(Ala401GlnfsTer8) | 11 | *De novo* | PVS1, PM2, PS2_mod | Pathogenic | NMD | MCR1 |
| Dillon et al.^34^ | L131 | c.1207C>T | g.33738611C>T | p.(Gln403Ter) | 11 | Unknown | PVS1, PM2 | Pathogenic | NMD | MCR1 |
| Bainbridge et al.^4^ | L72 | c.1210C>T | g.33738614C>T | p.(Gln404Ter) | 11 | *De novo* | PVS1, PM2, PS2_mod, PS4_mod | Pathogenic | NMD | MCR1 |
| Švantnerová et al.^35^ | L104 | c.1210C>T | g.33738614C>T | p.(Gln404Ter) | 11 | *De novo* | PVS1, PM2, PS2_mod, PS4_mod | Pathogenic | NMD | MCR1 |
| Woods et al.^5^ | U32 / F16a (our Family H) | c.1210C>T | g.33738614C>T | p.(Gln404Ter) | 11 | Apparent *de novo* | PVS1, PM2, PS2_mod, PS4_mod | Pathogenic | NMD | MCR1 |
| Woods et al.^5^ | U33 / F16b (our Family H) | c.1210C>T | g.33738614C>T | p.(Gln404Ter) | 11 | Apparent *de novo* | PVS1, PM2, PS2_mod, PS4_mod | Pathogenic | NMD | MCR1 |
| Kuechler et al.^14^ | L78 | c.1219del | g.33738623del | p.(Ser407AlafsTer2) | 11 | *De novo* | PVS1, PM2, PS2_mod | Pathogenic | NMD | MCR1 |
| Dad et al.^36^ | L63 | c.1314_1316delinsA | g.33738718_33738720delinsA | p.(Ser439ArgfsTer7) | 11 | *De novo* | PVS1, PM2, PM6_sup | Pathogenic | NMD | MCR1 |
| Bacrot et al.^37^ | L98 | c.1318dup | g.33738722dup | p.(Glu440GlyfsTer7) | 11 | *De novo* | PVS1, PM2, PS2_mod | Pathogenic | NMD | MCR1 |
| Kuechler et al.^14^ | L79 | c.1369G>T | g.33738773G>T | p.(Glu457Ter) | 11 | *De novo* | PVS1, PM2, PM6_sup | Pathogenic | NMD | MCR1 |
| Yu et al.^16^ | L95 | c.1377_1378del | g.33738781_33738782del | p.(Glu459AspfsTer2) | 11 | *De novo* | PVS1, PM2, PM6_sup | Pathogenic | NMD | MCR1 |
| Woods et al.^5^ | U6 | c.1378dup | g. 33738782dup | p.(Thr460AsnfsTer2) | 11 | *De novo* | PVS1, PM2, PS2_mod | Pathogenic | NMD | MCR1 |
| Valencia et al.^38^ | L137 | c.1384_1387dup | g.33738788_33738791dup | p.(Cys463TyrfsTer3) | 11 | *De novo* | PVS1, PM2, PM6_sup | Pathogenic | NMD | MCR1 |
| Powis et al.^39^ | L138 | c.1389_1390del | g.33738793_33738794del | p.(Cys463Ter) | 11 | *De novo* | PVS1, PM2, PM6_sup | Pathogenic | NMD | MCR1 |
| Bainbridge et al.^4^ | L73 | c.1396C>T | g.33738800C>T | p.(Gln466Ter) | 11 | *De novo* | PVS1, PM2, PS2_mod | Pathogenic | NMD | MCR1 |
| Schirwani et al.^3^ | L43 | c.1406_1409dup | g.33738810_33738813dup | p.(His470GlnfsTer3) | 11 | *De novo* | PVS1, PM2, PS2_mod | Pathogenic | NMD | MCR1 |
| Bainbridge et al.^4^ | L74 | c.1422dup | g.33738826dup | p.(Glu475Ter) | 11 | *De novo* | PVS1, PM2, PS2_mod | Pathogenic | NMD | MCR1 |
| Zheng et al.^40^ | L144 | c.1422_1446dup | g.33738826_33738850dup | p.(Leu483Ter) | 11 | *De novo* | PVS1, PM2, PM6_sup | Pathogenic | NMD | MCR1 |
| Pande et al.^11^ | L111 / F9a | c.1429dup | g.33738833dup | p.(Ser477PhefsTer2) | 11 | Apparent *de novo* | PVS1, PM2, PM6_sup | Pathogenic | NMD | MCR1 |
| Pande et al.^11^ | L112 / F9b | c.1429dup | g.33738833dup | p.(Ser477PhefsTer2) | 11 | Apparent *de novo* | PVS1, PM2, PM6_sup | Pathogenic | NMD | MCR1 |
| Srivastava et al.^2^ | L62 | c.1448dup | g.33738852dup | p.(Thr484AsnfsTer5) | 11 | *De novo* | PVS1, PM2, PS2_mod, PS4_sup | Pathogenic | NMD | MCR1 |
| Li et al.^41^ | L105 | c.1448dup | g.33738852dup | p.(Thr484AsnfsTer5) | 11 | *De novo* | PVS1, PM2, PS2_mod, PS4_sup | Pathogenic | NMD | MCR1 |
| Schirwani et al.^3^ | L19 | c.1451_1454del | g.33738855_33738858del | p.(Thr484IlefsTer8) | 11 | *De novo* | PVS1, PM2, PS2_mod | Pathogenic | NMD | MCR1 |
| Zhang et al.^42^ | L132 | c.1471C>T | g.33738875C>T | p.(Gln491Ter) | 11 | *De novo* | PVS1, PM2, PM6_sup, PS4_sup | Pathogenic | NMD | MCR1 |
| Balasubramanian et al.^32^ | L56 | c.1485_1488dup | g.33738889_33738892dup | p.(Asp497Ter) | 11 | *De novo* | PVS1, PM2, PS2_mod | Pathogenic | NMD | MCR1 |
| Balasubramanian et al.^32^ | L57 | c.1491dup | g.33738895dup | p.(Asn498Ter) | 11 | *De novo* | PVS1, PM2, PS2_mod | Pathogenic | NMD | MCR1 |
| Schirwani et al.^3^ | L32 | c.1500del | g.33738904del | p.(Glu500AspfsTer5) | 11 | *De novo* | PVS1, PM2, PS2_mod | Pathogenic | NMD | MCR1 |
| Schirwani et al.^3^ | L38 / PP2 | c.1505_1508dup | g.33738909_33738912dup | p.(Met504CysfsTer5) | 11 | *De novo* | PVS1, PM2, PS2_mod | Pathogenic | NMD | MCR1 |
| Schirwani et al.^3^ | L4 | c.1534_1535del | g.33738938_33738939del | p.(Leu512AlafsTer4) | 11 | *De novo* | PVS1, PM2, PS2_mod, PS4_sup | Pathogenic | NMD | MCR1 |
| Schirwani et al.^3^ | L16 | c.1534_1535del | g.33738938_33738939del | p.(Leu512AlafsTer4) | 11 | *De novo* | PVS1, PM2, PS2_mod, PS4_sup | Pathogenic | NMD | MCR1 |
| Schirwani et al.^3^ | L25 | c.1535T>A | g.33738939T>A | p.(Leu512Ter) | 11 | *De novo* | PVS1, PM2, PS2_mod | Pathogenic | NMD | MCR1 |
| Woods et al.^5^ | U28 | c.1550_1554del | g.33738954_33738958del | p.(Val517AspfsTer23) | 11 | *De novo* | PVS1, PM2, PS2_mod | Pathogenic | NMD | MCR1 |
| Arai et al.^43^ | L128 | c.1560_1569dup | g.33738964_33738973dup | p.(Glu524ArgfsTer21) | 11 | Unknown | PVS1, PM2 | Pathogenic | NMD | MCR1 |
| Woods et al.^5^ | U8 | c.1568C>G | g.33738972C>G | p.(Ser523Ter) | 11 | *De novo* | PVS1, PM2, PS2_mod | Pathogenic | NMD | MCR1 |
| Schirwani et al.^3^ | L24 | c.1579del | g.33738983del | p.(Gln527ArgfsTer4) | 11 | *De novo* | PVS1, PM2, PS2_mod | Pathogenic | NMD | MCR1 |
| Shirwani et al.^3^ | L17 | c.1579dup | g.33738983dup | p.(Gln527ProfsTer15) | 11 | *De novo* | PVS1, PM2, PS2_mod | Pathogenic | NMD | MCR1 |
| Schirwani et al.^3^ | L7 | c.1606C>T | g.33739010C>T | p.(Gln536Ter) | 11 | *De novo* | PVS1, PM2, PS2_mod | Pathogenic | NMD | MCR1 |
| Khan et al.^44^ | L89 | c.1612G>T | g.33739016G>T | p.(Glu538Ter) | 11 | *De novo* | PVS1, PM2, PM6_sup, PS4_sup | Pathogenic | NMD | MCR1 |
| Woods et al.^5^ | U26 | c.1612G>T | g.33739016G>T | p.(Glu538Ter) | 11 | Unknown | PVS1, PM2, PM6_sup, PS4_sup | Pathogenic | NMD | MCR1 |
| Taşkıran et al.^45^ | L148 | c.1627_1628del | g.33739031_33739032del | p.(Leu543TyrfsTer12) | 11 | Unknown | PVS1, PM2 | Pathogenic | NMD | MCR1 |
| Kim et al.^46^ | L159 | c.1628_1629del | g.33739032_33739033del | p.(Leu543HisfsTer12) | 11 | *De novo* | PVS1, PM2, PM6_sup | Pathogenic | NMD | MCR1 |
| Schirwani et al.^6^ | L103 | c.1632_1637delinsCTATGACTTCATCTATGACTCATGTCAGTGA (mosaic) | g.33739036_33739041delins31 | p.(Pro545TyrfsTer10) | 11 | *De novo* | PVS1, PM2, PS2_mod | Pathogenic | NMD | MCR1 |
| Woods et al.^5^ | U1 | c.1643C>A | g.33739047C>A | p.(Ser548Ter) | 11 | *De novo* | PVS1, PM2, PS2_mod | Pathogenic | NMD | MCR1 |
| Bowling et al.^47^ | L133 | c.1682C>A | g.33739086C>A | p.(Ser561Ter) | 11 | Unknown | PVS1, PM2 | Pathogenic | NMD | MCR1 |
| Schirwani et al.^3^ | L21 | c.1698_1699del | g.33739102_33739103del | p.(Glu566AspfsTer20) | 11 | *De novo* | PVS1, PM2, PS2_mod | Pathogenic | NMD | MCR1 |
| Wang et al.^48^ | L149 | c.1754del | g.33739158del | p.(Asn585MetfsTer6) | 11 | *De novo* | PVS1, PM2, PM6_sup | Pathogenic | NMD | MCR1 |
| Ziats et al.^49^ | L115 | c.1777G>T | g.33739181G>T | p.(Glu593Ter) | 11 | *De novo* | PVS1, PM2, PM6_sup | Pathogenic | NMD | MCR1 |
| Balasubramanian et al.^32^ | L50 | c.1783C>T | g.33739187C>T | p.(Gln595Ter) | 11 | *De novo* | PVS1, PM2, PS2_mod, PS4_sup | Pathogenic | NMD | MCR1 |
| Schirwani et al.^3^ | L28 | c.1783C>T | g.33739187C>T | p.(Gln595Ter) | 11 | *De novo* | PVS1, PM2, PS2_mod, PS4_sup | Pathogenic | NMD | MCR1 |
| Li et al.^50^ | L65 | c.1795G>T | g.33739199G>T | p.(Glu599Ter) | 11 | *De novo* | PVS1, PM2, PS2_mod | Pathogenic | NMD | MCR1 |
| Woods et al.^5^ | U27 | c.1801_1802del | g.33739205_33739206del | p.(Gln601AlafsTer9) | 11 | *De novo* | PVS1, PM2, PM6_sup | Pathogenic | NMD | MCR1 |
| Schirwani et al.^3^ | L14 | c.1884_1885del | g.33739288_33739289del | p.(Gly629ArgfsTer17) | 11 | *De novo* | PVS1, PM2, PM6_sup | Pathogenic | NMD | MCR1 |
| Dinwiddie et al.^52^ | L69 | c.1895dup | g.33739299dup | p.(Gln633ThrfsTer14) | 11 | *De novo* | PVS1, PM2, PM6_sup | Pathogenic | NMD | MCR1 |
| Dinwiddie et al.^52^ | L86 | c.1897_1898del | g.33739301_33739302del | p.(Gln633ValfsTer13) | 11 | *De novo* | PVS1, PM2, PS2_mod, PS4_mod | Pathogenic | NMD | MCR1 |
| Awamleh et al.^52^ | L145 | c.1897_1898del | g.33739301_33739302del | p.(Gln633ValfsTer13) | 11 | Unknown | PVS1, PM2, PS2_mod, PS4_mod | Pathogenic | NMD | MCR1 |
| Woods et al.^5^ | U20 | c.1897_1898del | g.33739301_33739302del | p.(Gln633ValfsTer13) | 11 | Unknown | PVS1, PM2, PS2_mod, PS4_mod | Pathogenic | NMD | MCR1 |
| Schirwani et al.^3^ | L8 | c.1921_1924dup | g.33739325_33739328dup | p.(Pro642HisfsTer6) | 11 | *De novo* | PVS1, PM2, PS2_mod | Pathogenic | NMD | MCR1 |
| Bainbridge et al.^4^ | L75 / PP12 | c.1978_1981del | g.33739382_33739385del | p.(Asp660AsnfsTer16) | 11 | *De novo* | PVS1, PM2, PS2_mod, PS4_sup | Pathogenic | NMD | MCR1 |
| Schirwani et al.^3^ | L71 | c.1990C>T | g.33739394C>T | p.(Gln664Ter) | 11 | *De novo* | PVS1, PM2, PS2_mod, PS4_mod | Pathogenic | NMD | MCR1 |
| Cuddapah et al.^53^ | L20 | c.1990C>T | g.33739394C>T | p.(Gln664Ter) | 11 | *De novo* | PVS1, PM2, PS2_mod, PS4_mod | Pathogenic | NMD | MCR1 |
| Woods et al.^5^ | U30 / F17 | c.1990C>T | g.33739394C>T | p.(Gln664Ter) | 11 | Maternal mosaicism | PVS1, PM2, PS2_mod, PS4_mod | Pathogenic | NMD | MCR1 |
| Woods et al.^5^ | U21 | c.2041_2042insA | g.33739445_33739446insA | p.(Ser681TyrfsTer8) | 11 | *De novo* | PVS1. PM2, PM6_sup | Pathogenic | NMD | MCR1 |
| Schirwani et al.^3^ | L3 | c.2066del | g.33739470del | p.(Ile689AsnfsTer12) | 11 | *De novo* | PVS1, PM2, PS2_mod | Pathogenic | NMD | MCR1 |
| Woods et al.^5^ | U31 / F18 | c.2070del | g.33739474del | p.(Glu691LysfsTer10) | 11 | Paternal mosaicism | PVS1, PM2 | Pathogenic | NMD | MCR1 |
| Guo et al.^54^ | L126 | c.2096del | g.33739500del | p.(Pro699HisfsTer2) | 11 | *De novo* | PVS1, PM2, PM6_sup | Pathogenic | NMD | MCR1 |
| Woods et al.^5^ | U25 | c.2237dup | g.33739641dup | p.(Leu746PhefsTer17) | 11 | *De novo* | PVS1, PM2, PM6_sup | Pathogenic | NMD | MCR1 |
| Schirwani et al.^3^ | L10 / F1a | c.2791_2792del | g.33740195_33740196del | p.(Gln931GlufsTer19) | 11 | Paternal | PVS1, PM2 | Pathogenic | NMD |  |
| Schirwani et al.^3^ | L11 / F1b | c.2791_2792del | g.33740195_33740196del | p.(Gln931GlufsTer19) | 11 | Unknown | PVS1, PM2 | Pathogenic | NMD |  |
| Woods et al.^5^ | U39 / F12a (our Family C) | c.2902G>T | g.33740306G>T | p.(Glu968Ter) | 11 | Maternal | PVS1, PM2 | Pathogenic | NMD |  |
| Woods et al.^5^ | U40 / F12b (our Family C) | c.2902G>T | g.33740306G>T | p.(Glu968Ter) | 11 | Unknown | PVS1, PM2 | Pathogenic | NMD |  |
| Contreras-Capetillo et al.^55^ | L85 | c.2992_2995del | g.33740396_33740399del | p.(Glu998LysfsTer26) | 11 | *De novo* | PVS1_str, PM2, PS2_mod | Likely pathogenic | no NMD |  |
| Wayhelova et al.^56^ | L87 | c.3006del | g.33740410del | p.(Arg1004GlufsTer21) | 11 | *De novo* | PVS1_str, PM2, PM6_sup | Likely pathogenic | no NMD |  |
| Chinen et al.^57^ | L84 | c.3032del | g.33740436del | p.(Pro1011LeufsTer14) | 11 | *De novo* | PVS1_str, PM2, PM6_sup | Likely pathogenic | no NMD |  |
| Tønne et al.^58^ | L154 | c.3033dup | g.33740437dup | p.(Leu1012SerfsTer23) | 11 | *De novo* | PVS1_str, PM2, PM6_sup | Likely pathogenic | no NMD |  |
| Myers et al.^59^ | L91 / PP4 | c.3039+1G>A | g.33740444G>A | Likely skipping of exon 11 | Intron 11 | *De novo* | PVS1_str, PM2, PS2_mod, PS4_mod, PS1 | Pathogenic | no NMD |  |
| Hori et al.^60^ | L97 | c.3039+1G>A | g.33740444G>A | Likely skipping of exon 11 | Intron 11 | *De novo* | PVS1_str, PM2, PS2_mod, PS4_mod, PS1 | Pathogenic | no NMD |  |
| Verberne et al.^61^ | L136 | c.3039+1G>T | g.33740444G>T | Likely skipping of exon 11 | Intron 11 | Unknown | PVS1_str, PM2, PS1 | Pathogenic | no NMD |  |
| Woods et al.^5^ | U12 | c.3039+2T>C | g.33740445T>C | Likely skipping of exon 11 | Intron 11 | *De novo* | PVS1_str, PM2, PS2_mod, PS1_mod | Pathogenic | no NMD |  |
| Wu et al.^62^ | L59 | c.3043C>T | g.33742891C>T | p.(Gln1015Ter) | 12 | *De novo* | PVS1_str, PM2, PS2_mod | Likely pathogenic | no NMD | MCR2 |
| Tie et al.^63^ | L142 | c.3096dup | g.33742944dup | p.(Pro1033ThrfsTer2) | 12 | *De novo* | PVS1_str, PM2, PM6_sup | Likely pathogenic | no NMD | MCR2 |
| Schirwani et al.^3^ | L76 / F7a | c.3106C>T | g.33742954C>T | p.(Arg1036Ter) | 12 | Unknown | PVS1_str, PM2, PS2_mod, PS4_mod | Pathogenic | no NMD | MCR2 |
| Koboldt et al.^9^ | L77 / F7b | c.3106C>T | g.33742954C>T | p.(Arg1036Ter) | 12 | Apparent *de novo* | PVS1_str, PM2, PS2_mod, PS4_mod | Pathogenic | no NMD | MCR2 |
| Koboldt et al.^9^ | L80 | c.3106C>T | g.33742954C>T | p.(Arg1036Ter) | 12 | Apparent *de novo* | PVS1_str, PM2, PS2_mod, PS4_mod | Pathogenic | no NMD | MCR2 |
| Kuechler et al.^14^ | L90 | c.3106C>T | g.33742954C>T | p.(Arg1036Ter) | 12 | *De novo* | PVS1_str, PM2, PS2_mod, PS4_mod | Pathogenic | no NMD | MCR2 |
| Myers et al.^59^ | L156 | c.3106C>T | g.33742954C>T | p.(Arg1036Ter) | 12 | *De novo* | PVS1_str, PM2, PS2_mod, PS4_mod | Pathogenic | no NMD | MCR2 |
| Heide et al.^64^ | L157 | c.3106C>T | g.33742954C>T | p.(Arg1036Ter) | 12 | *De novo* | PVS1_str, PM2, PS2_mod, PS4_mod | Pathogenic | no NMD | MCR2 |
| Duan et al.^65^ | L34 | c.3106C>T | g.33742954C>T | p.(Arg1036Ter) | 12 | *De novo* | PVS1_str, PM2, PS2_mod, PS4_mod | Pathogenic | no NMD | MCR2 |
| Woods et al.^5^ | U24 | c.3106C>T | g.33742954C>T | p.(Arg1036Ter) | 12 | *De novo* | PVS1_str, PM2, PS2_mod, PS4_mod | Pathogenic | no NMD | MCR2 |
| Balasubramanian et al.^32^ | L54 / PP13 | c.3127_3128dup | g.33742975_33742976dup | p.(Gly1045ValfsTer99) | 12 | *De novo* | PVS1_str, PM2, PS2_mod | Likely pathogenic | no NMD | MCR2 |
| Schirwani et al.^3^ | L30 | c.3178dup | g.33743026dup | p.(Arg1060ProfsTer50) | 12 | *De novo* | PVS1_str, PM2, PS2_mod, PS4_sup | Likely pathogenic | no NMD | MCR2 |
| Balasubramanian et al.^32^ | L55 | c.3178dup | g.33743026dup | p.(Arg1060ProfsTer50) | 12 | *De novo* | PVS1_str, PM2, PS2_mod, PS4_sup | Likely pathogenic | no NMD | MCR2 |
| Tie et al.^63^ | L143 | c.3253G>T | g.33743101G>T | p.(Gly1085Ter) | 12 | *De novo* | PVS1_str, PM2, PM6_sup | Likely pathogenic | no NMD | MCR2 |
| Schirwani et al.^6^ | L99 / F4a | c.3287_3291del | g.33743135_33743139del | p.(Thr1096AsnfsTer12) | 12 | Apparent *de novo* | PVS1_str, PM2, PS2_mod | Likely pathogenic | no NMD | MCR2 |
| Schirwani et al.^6^ | L100 / F4b | c.3287_3291del | g.33743135_33743139del | p.(Thr1096AsnfsTer12) | 12 | Apparent *de novo* | PVS1_str, PM2, PS2_mod | Likely pathogenic | no NMD | MCR2 |
| Yu et al.^16^ | L93 | c.3307A>T | g.33743155A>T | p.(Lys1103Ter) | 12 | *De novo* | PVS1_str, PM2, PM6_sup | Likely pathogenic | no NMD | MCR2 |
| Wang et al.^31^ | L92 | c.3315_3318del | g.33743163_33743166del | p.(Thr1106ArgfsTer36) | 12 | *De novo* | PVS1_str, PM2, PS2_mod | Likely pathogenic | no NMD | MCR2 |
| Yuan et al.^66^ | L150 | c.3325A>T | g.33743173A>T | p.(Lys1109Ter) | 12 | *De novo* | PVS1_str, PM2, PM6_sup | Likely pathogenic | no NMD | MCR2 |
| Woods et al.^5^ | U11 | c.3332_3333del | g.33743180_33743181del | p.(Phe1111CysfsTer14) | 12 | *De novo* | PVS1_str, PM2, PS2_mod | Likely pathogenic | no NMD | MCR2 |
| Cuddapah et al.^53^ | L70 | c.3349C>T | g.33743197C>T | p.(Arg1117Ter) | 12 | *De novo* | PVS1_str, PM2, PS2_mod, PS4_mod | Pathogenic | no NMD | MCR2 |
| Hegde et al.^67^ | L135 | c.3349C>T | g.33743197C>T | p.(Arg1117Ter) | 12 | *De novo* | PVS1_str, PM2, PS2_mod, PS4_mod | Pathogenic | no NMD | MCR2 |
| Zhang et al.^42^ | L158 | c.3349C>T | g.33743197C>T | p.(Arg1117Ter) | 12 | *De novo* | PVS1_str, PM2, PS2_mod, PS4_mod | Pathogenic | no NMD | MCR2 |
| Woods et al.^5^ | U10 | c.3349C>T | g.33743197C>T | p.(Arg1117Ter) | 12 | *De novo* | PVS1_str, PM2, PS2_mod, PS4_mod | Pathogenic | no NMD | MCR2 |
| Balasubramanian et al.^32^ | L51 / PP11 | c.3355dup | g.33743203dup | p.(His1119ProfsTer7) | 12 | *De novo* | PVS1_str, PM2, PS2_mod | Likely pathogenic | no NMD | MCR2 |
| Srivastavva et al.^2^ | L61 | c.3364C>T | g.33743212C>T | p.(Gln1122Ter) | 12 | *De novo* | PVS1_str, PM2, PS2_mod, PS4_mod | Pathogenic | no NMD | MCR2 |
| Hyder et al.^68^ | L152 | c.3364C>T | g.33743212C>T | p.(Gln1122Ter) | 12 | *De novo* | PVS1_str, PM2, PS2_mod, PS4_mod | Pathogenic | no NMD | MCR2 |
| Woods et al.^5^ | U16 | c.3364C>T | g.33743212C>T | p.(Gln1122Ter) | 12 | *De novo* | PVS1_str, PM2, PS2_mod, PS4_mod | Pathogenic | no NMD | MCR2 |
| Schirwani et al.^3^ | L12 | c.3382del | g.33743230del | p.(Arg1128GlyfsTer15) | 12 | *De novo* | PVS1_str, PM2, PM6_sup | Likely pathogenic | no NMD | MCR2 |
| Schirwani et al.^3^ | L42 | c.3419del | g.33743267del | p.(Pro1140GlnfsTer3) | 12 | *De novo* | PVS1_str, PM2, PS2_mod | Likely pathogenic | no NMD | MCR2 |
| Schirwani et al.^3^ | L33 | c.3464C>A | g.33743312C>A | p.(Ser1155Ter) | 12 | *De novo* | PVS1_str, PM2, PS2_mod, PS4_mod | Pathogenic | no NMD | MCR2 |
| Qiao et al.^69^ | L64 | c.3464C>A | g.33743312C>A | p.(Ser1155Ter) | 12 | *De novo* | PVS1_str, PM2, PS2_mod, PS4_mod | Pathogenic | no NMD | MCR2 |
| Yang et al.^70^ | L66 | c.3494_3495del | g.33743342_33743343del | p.(Cys1165Ter) | 12 | *De novo* | PVS1_str, PM2, PM6_sup, PS4_sup | Likely pathogenic | no NMD | MCR2 |
| Kuechler et al.^14^ | L81 | c.3494_3495del | g.33743342_33743343del | p.(Cys1165Ter) | 12 | *De novo* | PVS1_str, PM2, PM6_sup, PS4_sup | Likely pathogenic | no NMD | MCR2 |
| Ababneh et al.^71^ | L155 | c.3592_3593insGAT | g.33743440_33743441insGAT | p.(Leu1198Ter) | 12 | *De novo* | PVS1_str, PM2, PM6_sup | Likely pathogenic | no NMD | MCR2 |
| Woods et al.^5^ | U4 | c.3596_3612del | c.3596_3612del | p.(Ser1199Ter) | 12 | *De novo* | PVS1_str, PM2, PS2_mod | Likely pathogenic | no NMD | MCR2 |
| Kuechler et al.^14^ | L82 | c.3613G>T | g.33743461G>T | p.(Glu1205Ter) | 12 | *De novo* | PVS1_str, PM2, PS2_mod | Likely pathogenic | no NMD | MCR2 |
| Balasubramanian et al.^32^ | L53 | c.3635T>G | g.33743483T>G | p.(Leu1212Ter) | 12 | *De novo* | PVS1_str, PM2, PS2_mod | Likely pathogenic | no NMD | MCR2 |
| Chauhan et al.^72^ | L160 | c.3746_3749dup | g.33743594_33743597dup | p.(Lys1250AsnfsTer2) | 12 | *De novo* | PVS1_str, PM2, PM6_sup | Likely pathogenic | no NMD | MCR2 |
| Schirwani et al.^3^ | L6 | c.3811_3814dup | g.33743659_33743662dup | p.(Thr1272LysfsTer8) | 12 | *De novo* | PVS1_str, PM2, PS2_mod | Likely pathogenic | no NMD | MCR2 |
| Ayoub et al.^73^ | L106 | c.3964C>T | g.33743812C>T | p.(Gln1322Ter) | 12 | Unknown | PVS1_str, PM2 | Likely pathogenic | no NMD | MCR2 |
| Ayoub et al.^73^ | L110 / PP8 | c.4034_4035dup | g.33743882_33743883dup | p.(Ile1346ProfsTer15) | 12 | *De novo* | PVS1_str, PM2, PS2_mod | Likely pathogenic | no NMD | MCR2 |
| Ayoub et al.^73^ | L109 / PP6 | c.4060_4061del | g.33743908_33743909del | p.(Ser1354HisfsTer2) | 12 | *De novo* | PVS1_str, PM2, PS2_mod | Likely pathogenic | no NMD | MCR2 |
| Kuechler et al.^14^ | L83 | c.4072_4073del | g.33743920_33743921del | p.(Val1358LeufsTer8) | 12 | *De novo* | PVS1_str, PM2, PM6_sup | Likely pathogenic | no NMD | MCR2 |
| Ababneh et al.^71^ | L151 | c.4090G>T | g.33743938G>T | p.(Gly1364Ter) | 12 | *De novo* | PVS1_str, PM2, PM6_sup | Likely pathogenic | no NMD | MCR2 |
| Schirwani et al.^3^ | L40 | c.4120_4123dup | g.33743968_33743971dup | p.(Ala1375AspfsTer7) | 12 | *De novo* | PVS1_str, PM2, PS2_mod | Likely pathogenic | no NMD | MCR2 |
| Zhao et al.^74^ | L129 | c.4143dup | g.33743991dup | p.(Leu1395ProfsTer5) | 12 | *De novo* | PVS1_str, PM2, PS2_mod | Likely pathogenic | no NMD | MCR2 |
| Zhao et al.^74^ | L130 | c.4143dup | g.33743991dup | p.(Leu1395ProfsTer5) | 12 | *De novo* | PVS1_str, PM2, PS2_mod | Likely pathogenic | no NMD | MCR2 |
| Balasubramanian et al.^32^ | L49 | c.4144C>T | g.33743992C>T | p.(Gln1382Ter) | 12 | *De novo* | PVS1_str, PM2, PS2_mod | Likely pathogenic | no NMD | MCR2 |
| Zhu et al.^75^ | L153 | c.4153_4154del | g.33744001_33744002del | p.(Val1385IlefsTer14) | 12 | *De novo* | PVS1_str, PM2, PS2_mod | Likely pathogenic | no NMD | MCR2 |
| Woods et al.^5^ | U17 | c.4156del | g.33744004del | p.(Ser1386ProfsTer6) | 12 | Unknown | PVS1_str, PM2 | Likely pathogenic | no NMD | MCR2 |
| Guo et al.^54^ | L125 | c.4172_4173del | g.33744020_33744021del | p.(Val1391GlufsTer8) | 12 | *De novo* | PVS1_str, PM2, PM6_sup | Likely pathogenic | no NMD | MCR2 |
| Schirwani et al.^3^ | L39 | c.4219_4220del | g.33744067_33744068del | p.(Leu1407GlyfsTer20) | 12 | *De novo* | PVS1_str, PM2, PS2_mod, PS4_mod | Pathogenic | no NMD | MCR2 |
| Woods et al.^5^ | U18 | c.4219_4220del | g.33744067_33744068del | p.(Leu1407GlyfsTer20) | 12 | *De novo* | PVS1_str, PM2, PS2_mod, PS4_mod | Pathogenic | no NMD | MCR2 |
| Woods et al.^5^ | U23 | c.4219_4220del | g.33744067_33744068del | p.(Leu1407GlyfsTer20) | 12 | *De novo* | PVS1_str, PM2, PS2_mod, PS4_mod | Pathogenic | no NMD | MCR2 |
| Cuddapah et al.^53^ | L68 | c.4322C>G | g.33744170C>G | p.(Ser1441Ter) | 12 | *De novo* | PVS1_str, PM2, PM6_sup, PS4_sup | Likely pathogenic | no NMD | MCR2 |
| Woods et al.^5^ | U19 | c.4322C>G | g.33744170C>G | p.(Ser1441Ter) | 12 | *De novo* | PVS1_str, PM2, PM6_sup, PS4_sup | Likely pathogenic | no NMD | MCR2 |
| Schirwani et al.^3^ | L9 / PP10 | c.4330C>T | g.33744178C>T | p.(Arg1444Ter) | 12 | *De novo* | PVS1_str, PM2, PS2_mod PS4_mod | Pathogenic | no NMD | MCR2 |
| Schirwani et al.^3^ | L31 | c.4330C>T | g.33744178C>T | p.(Arg1444Ter) | 12 | *De novo* | PVS1_str, PM2, PS2_mod, PS4_mod | Pathogenic | no NMD | MCR2 |
| Balasubramanian et al.^32^ | L46 | c.4330C>T | g.33744178C>T | p.(Arg1444Ter) | 12 | *De novo* | PVS1_str, PM2, PS2_mod, PS4_mod | Pathogenic | no NMD | MCR2 |
| Srivastava et al.^2^ | L60 | c.4330C>T | g.33744178C>T | p.(Arg1444Ter) | 12 | Unknown | PVS1_str, PM2, PS2_mod PS4_mod | Pathogenic | no NMD | MCR2 |
| Fu et al.^76^ | L127 | c.4330C>T | g.33744178C>T | p.(Arg1444Ter) | 12 | Unknown | PVS1_str, PM2, PS2_mod PS4_mod | Pathogenic | no NMD | MCR2 |
| Schirwani et al.^3^ | L44 | c.4330C>T | g.33744178C>T | p.(Arg1444Ter) | 12 | *De novo* | PVS1_str, PM2, PS2_mod PS4_mod | Pathogenic | no NMD | MCR2 |
| Woods et al.^5^ | U2 | c.4330C>T | g.33744178C>T | p.(Arg1444Ter) | 12 | *De novo* | PVS1_str, PM2, PS2_mod, PS4_mod | Pathogenic | no NMD | MCR2 |
| Schirwani et al.^3^ | U29 | c.4330C>T | g.33744178C>T | p.(Arg1444Ter) | 12 | *De novo* | PVS1_str, PM2, PS2_mod, PS4_mod | Pathogenic | no NMD | MCR2 |
| Schirwani et al.^3^ | L41 | c.4336_4337del | g.33744184_33744185del | p.(Arg1446GlyfsTer3) | 12 | *De novo* | PVS1_str, PM2, PS2_mod, PS4_sup | Likely pathogenic | no NMD | MCR2 |
| Schirwani et al.^3^ | L35 | c.4360C>T | g.33744208C>T | p.(Gln1454Ter) | 12 | *De novo* | PVS1_str, PM2, PS2_mod, PS4_sup | Likely pathogenic | no NMD | MCR2 |
| Hu et al.^77^ | L116 | c.4360C>T | g.33744208C>T | p.(Gln1454Ter) | 12 | *De novo* | PVS1_str, PM2, PS2_mod, PS4_sup | Likely pathogenic | no NMD | MCR2 |
| Schirwani et al.^3^ | L26 | c.4399C>T | g.33744247C>T | p.(Arg1467Ter) | 12 | *De novo* | PVS1_str, PS2_mod, PS4_mod | Likely pathogenic | no NMD | MCR2 |
| Schirwani et al.^3^ | L27 | c.4399C>T | g.33744247C>T | p.(Arg1467Ter) | 12 | Unknown | PVS1_str, PS2_mod, PS4_mod | Likely pathogenic | no NMD | MCR2 |
| Schirwani et al.^3^ | L36 | c.4399C>T | g.33744247C>T | p.(Arg1467Ter) | 12 | *De novo* | PVS1_str, PS2_mod, PS4_mod | Likely pathogenic | no NMD | MCR2 |
| Yu et al.^16^ | L96 | c.4399C>T | g.33744247C>T | p.(Arg1467Ter) | 12 | *De novo* | PVS1_str, PS2_mod, PS4_mod | Likely pathogenic | no NMD | MCR2 |
| Woods et al.^5^ | U15 | c.4399C>T | g.33744247C>T | p.(Arg1467Ter) | 12 | *De novo* | PVS1_str, PS2_mod, PS4_mod | Likely pathogenic | no NMD | MCR2 |
| Hu et al.^77^ | L117 | c.4400_4403dup | g.33744248_33744251dup | p.(Pro1470AsnfsTer4) | 12 | *De novo* | PVS1_str, PM2, PM6_sup | Likely pathogenic | no NMD | MCR2 |
| Schirwani et al.^3^ | L22 / F3 | c.4441dup | g.33744289dup | p.(Leu1481ProfsTer12) | 12 | Maternal | PVS1_str, PM2 | Likely pathogenic | no NMD | MCR2 |
| Schirwani et al.^3^ | L23 / PP9 | c.4462_4465del | g.33744310_33744313del | p.(Thr1488SerfsTer17) | 12 | *De novo* | PVS1_str, PM2, PS2_mod, PS4_sup | Likely pathogenic | no NMD | MCR2 |
| Bartolomaeus et al.^10^ | L122 / F8a | c.4462_4465del | g.33744310_33744313del | p.(Thr1488SerfsTer17) | 12 | Paternal | PVS1_str, PM2, PS2_mod, PS4_sup | Likely pathogenic | no NMD | MCR2 |
| Bartolomaeus et al.^10^ | L123 / F8b | c.4462_4465del | g.33744310_33744313del | p.(Thr1488SerfsTer17) | 12 | Paternal | PVS1_str, PM2, PS2_mod, PS4_sup | Likely pathogenic | no NMD | MCR2 |
| Schirwani et al.^3^ | L1 / PP5 | c.4479_4483del | g.33744327_33744331del | p.(Ser1493ArgfsTer29) | 12 | *De novo* | PVS1_str, PM2, PS2_mod | Likely pathogenic | no NMD | MCR2 |
| Schirwani et al.^6^ | L101 / F5a | c.4509_4513dup | g.33744357_33744361dup | p.(Val1505AspfsTer3) | 12 | Apparent *de novo* | PVS1_str, PM2, PS2_mod | Likely pathogenic | no NMD | MCR2 |
| Schirwani et al.^6^ | L102 / F5b | c.4509_4513dup | g.33744357_33744361dup | p.(Val1505AspfsTer3) | 12 | Apparent *de novo* | PVS1_str, PM2, PS2_mod | Likely pathogenic | no NMD | MCR2 |
| Schirwani et al.^3^ | L2 / F2a | c.4534C>T | g.33744382C>T | p.(Gln1512Ter) | 12 | Maternal | PVS1_str, PM2, PS2_mod, PS4_sup | Likely pathogenic | no NMD | MCR2 |
| Schirwani et al.^3^ | L113 / F2b | c.4534C>T | g.33744382C>T | p.(Gln1512Ter) | 12 | *De novo* | PVS1_str, PM2, PS2_mod, PS4_sup | Likely pathogenic | no NMD | MCR2 |
| Schirwani et al.^7^ | L114 / F2c | c.4534C>T | g.33744382C>T | p.(Gln1512Ter) | 12 | Maternal | PVS1_str, PM2, PS2_mod, PS4_sup | Likely pathogenic | no NMD | MCR2 |
| Schirwani et al.^7^ | L37 | c.4534C>T | g.33744382C>T | p.(Gln1512Ter) | 12 | *De novo* | PVS1_str, PM2, PS2_mod, PS4_sup | Likely pathogenic | no NMD | MCR2 |
| Woods et al.^5^ | U41 / F13a (our Family D) | c.4611del | g.33744459del | p.(Thr1538LeufsTer32) | 12 | Paternal | PVS1_str, PM2 | Likely pathogenic | no NMD | MCR2 |
| Woods et al.^5^ | U42 / F13b (our Family D) | c.4611del | g.33744459del | p.(Thr1538LeufsTer32) | 12 | Paternal | PVS1_str, PM2 | Likely pathogenic | no NMD | MCR2 |
| Woods et al.^5^ | U43 / F13c (our Family D) | c.4611del | g.33744459del | p.(Thr1538LeufsTer32) | 12 | Unknown | PVS1_str, PM2 | Likely pathogenic | no NMD | MCR2 |
| Woods et al.^5^ | U44 / F13d (our Family D) | c.4611del | g.33744459del | p.(Thr1538LeufsTer32) | 12 | Paternal | PVS1_str, PM2 | Likely pathogenic | no NMD | MCR2 |
| Ayoub et al.^73^ | L108 | c.4648A>T | g.33744496A>T | p.(Lys1550Ter) | 12 | Unknown | PVS1_str, PM2 | Likely pathogenic | no NMD | MCR2 |
| Slatnick et al.^78^ | L118 | c.4678C>T | g.33744526C>T | p.(Arg1560Ter) | 12 | *De novo* | PVS1_str, PS2_mod | Likely pathogenic | no NMD | MCR2 |
| Nagy et al.^8^ | L119 / F6a | c.4678C>T | g.33744526C>T | p.(Arg1560Ter) | 12 | Maternal | PVS1_str, PS2_mod | Likely pathogenic | no NMD | MCR2 |
| Nagy et al.^8^ | L120 / F6b | c.4678C>T | g.33744526C>T | p.(Arg1560Ter) | 12 | Unknown | PVS1_str, PS2_mod | Likely pathogenic | no NMD | MCR2 |
| Naralan et al.^79^ | L121 | c.4678C>T | g.33744526C>T | p.(Arg1560Ter) | 12 | *De novo* | PVS1_str, PS2_mod | Likely pathogenic | no NMD | MCR2 |
| Woods et al.^5^ | U3 | c.4744C>T | g.33744592C>T | p.(Gln1582Ter) | 12 | *De novo* | PVS1_str, PM2, PS2_mod | Likely pathogenic | no NMD | MCR2 |
| Ayoub et al.^73^ | L107 / PP7 | c.4788_4816delinsT | g.33744636_33744664delinsT | p.(Cys1597GlyfsTer9) | 12 | *De novo* | PVS1_str, PM2, PS2_mod | Likely pathogenic | no NMD | MCR2 |
| Bonini et al.^80^ | L139 | c.4826G>A | g.33744674G>A | p.(Trp1609Ter) | 12 | *De novo* | PVS1_str, PM2, PM6_sup | Likely pathogenic | no NMD | MCR2 |
| Schirwani et al.^3^ | L29 / PP3 | c.4871_4874del | g.33744719_33744722del | p.(His1624ArgfsTer10) | 12 | *De novo* | PVS1_str, PM2, PS2_mod | Likely pathogenic | no NMD | MCR2 |
| Schirwani et al.^3^ | L18 | c.4894_4895del | g.33744742_33744743del | p.(Glu1632IlefsTer13) | 12 | *De novo* | PVS1_str, PM2, PS2_mod | Likely pathogenic | no NMD | MCR2 |
| Woods et al.^5^ | U13 | c.4899T>A | g.33744747T>A | p.(Tyr1633Ter) | 12 | *De novo* | PVS1_str, PM2, PS2_mod | Likely pathogenic | no NMD | MCR2 |
| Awamleh et al.^52^ | L146 | c.4906C>T | g.33744754C>T | p.(Gln1636Ter) | 12 | Unknown | PVS1_str, PM2 | Likely pathogenic | no NMD | MCR2 |
| Yu et al.^16^ | L94 | c.5455C>T | g.33745303C>T | p.(Gln1819Ter) | 12 | *De novo* | PVS1_str, PM2, PM6_sup | Likely pathogenic | no NMD |  |
| Guo et al.^54^ | L124 | c.5467C>T | g.33745315C>T | p.(Arg1823Ter) | 12 | *De novo* | PVS1_str, PM2, PM6_sup | Likely pathogenic | no NMD |  |
| Schirwani et al.^3^ | L5 / PP1 | c.5659A>T | g.33745507A>T | p.(Arg1887Ter) | 12 | *De novo* | PVS1_str, PM2, PS2_mod | Likely pathogenic | no NMD |  |
| Woods et al.^5^ | U14 | c.6110dup | g.33745958dup | p.(Pro2038ThrfsTer10) | 12 | *De novo* | PVS1_mod, PS2_mod, PP4 | Likely pathogenic | no NMD |  |
| Woods et al.^5^ | U45 / F14a (our Family E) | c.6199_6202del | g.33746047_33746050del | p.(Leu2067ValfsTer12) | 12 | Maternal | PVS1_mod, PM2, PS2_mod | Likely pathogenic | no NMD |  |
| Woods et al.^5^ | U46 / F14b (our Family E) | c.6199_6202del | g.33746047_33746050del | p.(Leu2067ValfsTer12) | 12 | *De novo* | PVS1_mod, PM2, PS2_mod | Likely pathogenic | no NMD |  |
| Verhoeven et al.^23^ | L88 | c.6697_6710dup | g.33746545_33746558dup | p.(Ser2238ThrfsTer3) | 12 | Unknown | PVS1_mod, PM2 | VUS | no NMD |  |
| Woods et al.^5^ | U37 / F11a (our Family B) | 18q12.1(32218967_33612863)×1 | g.32218967_33612863del | Start loss | 1-2 | Paternal | CNV guidelines - 1A, 2C-1 (0.9), 3A – total 0.9 | Likely pathogenic | NMD |  |
| Woods et al.^5^ | U38 / F11b (our Family B) | 18q12.1(32218967_33612863)×1 | g.32218967_33612863del | Start loss | 1-2 | Unknown | CNV guidelines - 1A, 2C-1 (0.9), 3A – total 0.9 | Likely pathogenic | NMD |  |
| Woods et al.^5^ | U9 | 18q12.1q12.3(25897038_37400276)×1 | g.25897038_374002766del | Multi-gene deletion | 1-12 | Unknown | CNV guidelines – 1A, 2A (1), 3C (0.9), 5F – total 1.9 | Pathogenic | NMD |  |
| Schirwani et al.^3^ | L15 | c.54+13836_880-552del (c.55_879del) | g.33592521_33731416del | p.(Ala19_Gln293del) | 2-8 | *De novo* | PVS1_Str, PM2, PS2_mod | Likely pathogenic | no NMD |  |
| Woods et al.^5^ | U22 | 18q12.1(33687447_33846149)×1 | g.33687447_33846149del | Multi-exon deletion | 9-12 | Unknown | CNV guidelines – 1A, 2D-4 (1), 3A, 5F – total 1 | Pathogenic | NMD |  |
| Woods et al.^5^ | U47 / F15a (our Family F) | 18q12.1(33697293_33839862)×1 | g.33697293_33839862del | Multi-exon deletion | 9-12 | Maternal | CNV guidelines – 1A, 2D-4 (1), 3A, 5D (0) – total 1 | Pathogenic | NMD |  |
| Woods et al.^5^ | U48 / F15b (our Family F) | 18q12.1(33697293_33839862)×1 | g.33697293_33839862del | Multi-exon deletion | 9-12 | Unknown | CNV guidelines – 1A, 2D-4 (1), 3A, 5D (0) – total 1 | Pathogenic | NMD |  |

**Supplementary Material 1** - List of individuals included in the genotype-phenotype correlation analysis, their ASXL3 variant and consequence, the classification of the variant and the assigned grouping for analysis (NMD/no NMD and MCR1/MCR2). Patients were assigned an ID number which denotes whether they were obtained from the literature or personal communication and whether they are part of a family. L = obtained from the literature, PP = previously published but updated clinical details reassessed as part of the Natural History Study, U = previously unpublished before Woods et al.^5^, F=family. All variants are described using HGVS nomenclature, reference transcript NM_030632.3.

References

31. Wang Q., Zhang J., Jiang N., Xie J., Yang J., Zhao X. De novo nonsense variant in ASXL3 in a Chinese girl causing Bainbridge-Ropers syndrome: a case report and review of literature. Mol Genet Genomic Med. 2022;10(5):e1924. doi:10.1002/mgg3.1924.

32. Balasubramanian M., Willoughby J., Fry A.E., et al. Delineating the phenotypic spectrum of Bainbridge-Ropers syndrome: 12 new patients with de novo, heterozygous, loss-of-function mutations in ASXL3 and review of published literature. J Med Genet. 2017;54(8):537–543. doi:10.1136/jmedgenet-2016-104360.

33. Lee H., Huang A.Y., Wang L.K., et al. Diagnostic utility of transcriptome sequencing for rare Mendelian diseases. Genet Med. 2020;22(3):490–499. doi:10.1038/s41436-019-0672-1.

34. Dillon O.J., Lunke S., Stark Z., et al. Exome sequencing has higher diagnostic yield compared to simulated disease-specific panels in children with suspected monogenic disorders. Eur J Hum Genet. 2018;26(5):644–651. doi:10.1038/s41431-018-0099-1.

35. Švantnerová J., Minár M., Radová S., Kolníková M., Vlkovič P., Zech M. ASXL3 de novo variant-related neurodevelopmental disorder presenting as dystonic cerebral palsy. Neuropediatrics. 2022;53(5):361–365. doi:10.1055/s-0042-1750721.

36. Dad R., Walker S., Scherer S.W., Hassan M.J., Kang S.Y., Minassian B.A. Hyperventilation-athetosis in ASXL3 deficiency (Bainbridge-Ropers) syndrome. Neurol Genet. 2017;3(5):e189. doi:10.1212/NXG.0000000000000189.

37. Bacrot S., Mechler C., Talhi N., et al. Whole exome sequencing diagnoses the first fetal case of Bainbridge-Ropers syndrome presenting as pontocerebellar hypoplasia type 1. Birth Defects Res. 2018;110(6):538–542. doi:10.1002/bdr2.1191.

38. Valencia C.A., Husami A., Holle J., et al. Clinical impact and cost-effectiveness of whole exome sequencing as a diagnostic tool: a pediatric Center’s experience. Front Pediatr. 2015;3:67. doi:10.3389/fped.2015.00067.

39. Powis Z., Farwell Hagman K.D., Blanco K., et al. When moments matter: finding answers with rapid exome sequencing. Mol Genet Genomic Med. 2020;8(2):e1027. doi:10.1002/mgg3.1027.

40. Zheng S., Chen H., Mo M. A case of Bainbridge-Ropers syndrome with autism in conjunct with ASXL3 gene variant and its clinical analysis. Zhonghua Yi Xue Yi Chuan Xue Za Zhi. 2021;38(7):671–673. doi:10.3760/cma.j.cn511374-20200901-00642.

41. Li J., Xu J., She M., Shi P., Kong X., Wang L. Genetic analysis and prenatal diagnosis for a Chinese pedigree affected with Bainbridge-Ropers syndrome. Zhonghua Yi Xue Yi Chuan Xue Za Zhi. 2022;39(11):1228–1232. doi:10.3760/cma.j.cn511374-20210813-00667.

42. Zhang H., Chen X., Tan H., et al. The exploration of genetic aetiology and diagnostic strategy for 321 Chinese individuals with intellectual disability. Clin Chim Acta. 2023;538:94–103. doi:10.1016/j.cca.2022.10.023.

43. Arai Y., Okanishi T., Okazaki T., et al. An adolescent case of ASXL3-related disorder with delayed onset of feeding difficulty. BMC Pediatr. 2024;24(1):308. doi:10.1186/s12887-024-04774-3.

44. Khan T.R., Dolce A., Goodspeed K. A case of Bainbridge-Ropers syndrome with breath holding spells and intractable epilepsy: challenges in diagnosis and management. BMC Neurol. 2022;22(1):60. doi:10.1186/s12883-022-02573-w.

45. Taşkıran E.Z., Karaosmanoğlu B., Koşukcu C., et al. Diagnostic yield of whole-exome sequencing in non-syndromic intellectual disability. J Intellect Disabil Res. 2021;65(6):577–588. doi:10.1111/jir.12835.

46. Kim J., Lee J., Jang D.H. Combining chromosomal microarray and clinical exome sequencing for genetic diagnosis of intellectual disability. Sci Rep. 2023;13(1):22807. doi:10.1038/s41598-023-50285-z.

47. Bowling K.M., Thompson M.L., Amaral M.D., et al. Genomic diagnosis for children with intellectual disability and/or developmental delay. Genome Med. 2017;9(1):43. doi:10.1186/s13073-017-0433-1.

48. Wang C., Zhou W., Zhang L., et al. Diagnostic yield and novel candidate genes for neurodevelopmental disorders by exome sequencing in an unselected cohort with microcephaly. BMC Genomics. 2023;24(1):422. doi:10.1186/s12864-023-09505-z.

49. Ziats M.N., Ahmad A., Bernat J.A., et al. Genotype-phenotype analysis of 523 patients by genetics evaluation and clinical exome sequencing. Pediatr Res. 2020;87(4):735–739. doi:10.1038/s41390-019-0611-5.

50. Li J.R., Huang Z., Lu Y., Ji Q.Y., Jiang M.Y., Yang F. Novel mutation in the ASXL3 gene in a Chinese boy with microcephaly and speech impairment: a case report. World J Clin Cases. 2020;8(24):6465–6472. doi:10.12998/wjcc.v8.i24.6465.

51. Dinwiddie D.L., Soden S.E., Saunders C.J., et al. De novo frameshift mutation in ASXL3 in a patient with global developmental delay, microcephaly, and craniofacial anomalies. BMC Med Genomics. 2013;6:32. doi:10.1186/1755-8794-6-32.

52. Awamleh Z., Chater-Diehl E., Choufani S., et al. DNA methylation signature associated with Bohring-Opitz syndrome: a new tool for functional classification of variants in ASXL genes. Eur J Hum Genet. 2022;30(6):695–702. doi:10.1038/s41431-022-01083-0.

53. Cuddapah V.A., Dubbs H.A., Adang L., et al. Understanding the phenotypic spectrum of ASXL-related disease: ten cases and a review of the literature. Am J Med Genet A. 2021;185(6):1700–1711. doi:10.1002/ajmg.a.62156.

54. Guo H., Wang T., Wu H., et al. Inherited and multiple de novo mutations in autism/developmental delay risk genes suggest a multifactorial model. Mol Autism. 2018;9:64. doi:10.1186/s13229-018-0247-z.

55. Contreras-Capetillo S.N., Vilchis-Zapata Z.H., Ribbón-Conde J., Pinto-Escalante D. Global developmental delay and postnatal microcephaly: Bainbridge-Ropers syndrome with a new mutation in ASXL3. Neurol (Engl Ed). 2018;33(7):484–486. doi:10.1016/j.nrl.2017.01.022.

56. Wayhelova M., Oppelt J., Smetana J., et al. Novel de novo frameshift variant in the ASXL3 gene in a child with microcephaly and global developmental delay. Mol Med Rep. 2019;20(1):505–512. doi:10.3892/mmr.2019.10303.

57. Chinen Y., Nakamura S., Ganaha A., et al. Mild prominence of the Sylvian fissure in a Bainbridge-Ropers syndrome patient with a novel frameshift variant in ASXL3. Clin Case Rep. 2018;6(2):330–336. doi:10.1002/ccr3.1361.

58. Tønne E., Due-Tønnessen B.J., Mero I.L., et al. Benefits of clinical criteria and high-throughput sequencing for diagnosing children with syndromic craniosynostosis. Eur J Hum Genet. 2021;29(6):920–929. doi:10.1038/s41431-020-00788-4.

59. Myers K.A., White S.M., Mohammed S., et al. Childhood-onset generalized epilepsy in Bainbridge-Ropers syndrome. Epilepsy Res. 2018;140:166–170. doi:10.1016/j.eplepsyres.2018.01.014.

60. Hori I., Miya F., Ohashi K., et al. Novel splicing mutation in the ASXL3 gene causing Bainbridge-Ropers syndrome. Am J Med Genet A. 2016;170(7):1863–1867. doi:10.1002/ajmg.a.37653.

61. Verberne E.A., Westermann J.M., de Vries T.I., et al. Genetic care in geographically isolated small island communities: 8 years of experience in the Dutch Caribbean. Am J Med Genet A. 2022;188(6):1777–1791. doi:10.1002/ajmg.a.62708.

62. Wu K., Cong Y. Case report: a novel ASXL3 gene variant in a Sudanese boy. BMC Pediatr. 2021;21(1):557. doi:10.1186/s12887-021-03038-8.

63. Tie X., Yang Y., He C., Zhang L., Che F. [Analysis of clinical feature and genetic variants in two Chinese pedigrees affected with Bainbridge-Ropers syndrome]. Zhonghua Yi Xue Yi Chuan Xue Za Zhi. 2022;39(8):836–841. doi:10.3760/cma.j.cn511374-20210702-00561.

64. Heide S., Spentchian M., Valence S., et al. Prenatal exome sequencing in 65 fetuses with abnormality of the corpus callosum: contribution to further diagnostic delineation. Genet Med. 2020;22(11):1887–1891. doi:10.1038/s41436-020-0872-8.

65. Duan F., Zhai Y., Kong X. Analysis of ASXL3 gene variant in a child with Bainbridge-Ropers syndrome. Zhonghua Yi Xue Yi Chuan Xue Za Zhi. 2021;38(3):275–277. doi:10.3760/cma.j.cn511374-20200219-00089.

66. Yuan B., Neira J., Pehlivan D., et al. Clinical exome sequencing reveals locus heterogeneity and phenotypic variability of cohesinopathies. Genet Med. 2019;21(3):663–675. doi:10.1038/s41436-018-0085-6.

67. Hegde M., Santani A., Mao R., Ferreira-Gonzalez A., Weck K.E., Voelkerding K.V. Development and validation of clinical whole-exome and whole-genome sequencing for detection of germline variants in inherited disease. Arch Pathol Lab Med. 2017;141(6):798–805. doi:10.5858/arpa.2016-0622-RA.

68. Hyder Z., Calpena E., Pei Y., et al. Evaluating the performance of a clinical genome sequencing program for diagnosis of rare genetic disease, seen through the lens of craniosynostosis. Genet Med. 2021;23(12):2360–2368. doi:10.1038/s41436-021-01297-5.

69. Qiao L., Liu Y., Ge J., Li T. Novel Nonsense Mutation in ASXL3 causing Bainbridge-Ropers Syndrome. Indian Pediatr. 2019;56(9):792–794. doi:10.1007/s13312-019-1627-y.

70. Yang L., Guo B., Zhu W., et al. Bainbridge-Ropers syndrome caused by loss-of-function variants in ASXL3: clinical abnormalities, medical imaging features, and gene variation in infancy of case report. BMC Pediatr. 2020;20(1):287. doi:10.1186/s12887-020-02027-7.

71. Ababneh F., Nashabat M., Alfadhel M. A new case of Bainbridge-Ropers syndrome (BRPS): delineating the phenotype and review of literature. J Biochem Clin Genet. 2019;2(1):65–69. doi:10.24911/JBCGenetics/183-1532439299.

72. Chauhan S., Arora V., Kulshrestha S., Suman P., Mushtaq I., Kumar P. First Report of Bainbridge-Ropers Syndrome in an Indian Individual. <https://iamg.in/genetic_clinics/full_text3bb2.html?id=388>. [Accessed 3 April 2025].

73. Ayoub M.C., Anderson J.T., Russell B.E., Wilson R.B. Examining the neurodevelopmental and motor phenotypes of Bohring-Opitz syndrome (ASXL1) and Bainbridge-Ropers syndrome (ASXL3). Front Neurosci. 2023;17:1244176. doi:10.3389/fnins.2023.1244176.

74. Zhao J.J., Halvardson J., Zander C.S., et al. Exome sequencing reveals NAA15 and PUF60 as candidate genes associated with intellectual disability. Am J Med Genet B Neuropsychiatr Genet. 2018;177(1):10–20. doi:10.1002/ajmg.b.32574.

75. Zhu X., Petrovski S., Xie P., et al. Whole-exome sequencing in undiagnosed genetic diseases: interpreting 119 trios. Genet Med. 2015;17(10):774–781. doi:10.1038/gim.2014.191.

76. Fu C., Luo S., Zhang Y., et al. Chromosomal microarray and whole exome sequencing identify genetic causes of congenital hypothyroidism with extra-thyroidal congenital malformations. Clin Chim Acta. 2019;489:103–108. doi:10.1016/j.cca.2018.11.035.

77. Hu C., Wang Y., Li C., et al. Targeted sequencing and clinical strategies in children with autism spectrum disorder: a cohort study. Front Genet. 2023;14:1083779. doi:10.3389/fgene.2023.1083779.

78. Slatnick L.R., Angione K., Hartman L. Precursor B-cell acute lymphoblastic leukemia in a pediatric patient with Bainbridge-Ropers syndrome. Pediatr Blood Cancer. 2023;70(1):e29873. doi:10.1002/pbc.29873.

79. Sümeyra Naralan Y., Enes Naralan M., Hocaoğlu Ç. Bainbridge Ropers syndrome as a rare cause of autism spectrum disorder. Psychiatr Danub. 2023;35(4):582–586. doi:10.24869/psyd.2023.582.

80. Bonini K.E., Thomas-Wilson A., Marathe P.N., et al. Identification of copy number variants with genome sequencing: clinical experiences from the NYCKidSeq program. Clin Genet. 2023;104(2):210–225. doi:10.1111/cge.14365.
