# Supplementary material for: Broadening the inherited ASXL3 spectrum and unveiling molecular mechanisms through detailed genotypic-phenotypic analyses
Source: Genet Med Open. 2026 May 28;4:104409. doi: 10.1016/j.gimo.2026.104409 (PMC13393785; doi:10.1016/j.gimo.2026.104409)
Supplement: Supplemental Material 2 [file mmc2.docx]

Supplementary Material 2 - Pearson chi-squared analysis for variant distribution

Supplementary Material 2 – Statistical analysis performed on *ASXL3* variants observed in patients and controls to assess whether variants are distributed evenly across the gene. Each variant was counted once, ignoring multiple occurrences. Analysis was performed on the gene as a whole, assessing the observed vs. expected number of variants for each exon, for the patient variants, and control variants excluding variant calls which were deemed to be of insufficient quality or miscalled as LOF. In all cases, variants were not distributed evenly, relative to the size of each exon. Exons 11 and 12 were then assessed individually. In the patient cohort, variants were clustered in exon 11 more than expected for its size, while in the control cohort, there were fewer variants in exon 11 than would be expected for its size. Exon 12 harboured a proportionate number of variants in patient and control cohorts. Following the exclusion of poor quality and non-LOF variants, the number of variants in both exon 11 and exon 12 was lower than expected.
