# Supplementary material for: Broadening the inherited ASXL3 spectrum and unveiling molecular mechanisms through detailed genotypic-phenotypic analyses
Source: Genet Med Open. 2026 May 28;4:104409. doi: 10.1016/j.gimo.2026.104409 (PMC13393785; doi:10.1016/j.gimo.2026.104409)
Supplement: Supplemental Material 3 [file mmc3.docx]

|  |  | **NMD** | | **no NMD** | | ***p =*** |  | **MCR1** | | **MCR2** | | ***p =*** |
| --- | --- | --- | --- | --- | --- | --- | --- | --- | --- | --- | --- | --- |
| **PREGNANCY AND BIRTH** | Pregnancy complications | 23/61 | 37.70% | 29/72 | 40.28% | **0.1353** |  | 18/45 | 40.00% | 24/61 | 39.34% | **0.1586** |
|  | C-Section | 25/52 | 48.08% | 34/72 | 47.22% | **0.1438** |  | 18/38 | 47.37% | 28/60 | 46.67% | **0.1640** |
|  | Admitted to NICU | 23/59 | 38.98% | 31/72 | 43.06% | **0.1422** |  | 21/45 | 46.67% | 26/61 | 42.62% | **0.1438** |
|  | Weeks of gestation | 38.88 |  | 39.01 |  | **0.6563** |  | 38.98 |  | 39.03 |  | **0.8843** |
|  | Birth weight (SD) | -0.23 |  | -0.29 |  | **0.7452** |  | -0.23 |  | -0.27 |  | **0.8362** |
|  | Birth OFC (SD) | -0.17 |  | 0.37 |  | **0.1571** |  | -0.10 |  | 0.48 |  | **0.1595** |
| **GROWTH PARAMETERS** | Follow-up weight (SD) | -1.39 |  | -1.67 |  | **0.4649** |  | -1.83 |  | -1.60 |  | **0.5892** |
|  | Follow-up height (SD) | -1.47 |  | -1.55 |  | **0.8079** |  | -1.77 |  | -1.48 |  | **0.4371** |
|  | Follow-up OFC (SD) | -2.56 |  | -1.69 |  | **0.0310** |  | -2.70 |  | -1.55 |  | **0.0085** |
|  | Difference in weight (SD) | -1.17 |  | -1.40 |  | **0.5552** |  | -1.53 |  | -1.36 |  | **0.6872** |
|  | Difference in OFC (SD) | -2.46 |  | -2.15 |  | **0.6019** |  | -2.90 |  | -1.94 |  | **0.0959** |
|  | Microcephaly | 40/68 | 58.82% | 40/88 | 45.45% | **0.0330** |  | 36/55 | 65.45% | 34/75 | 45.33% | **0.0109** |
| **DEVELOPMENTAL PARAMETERS** | Sitting independently (months) | 12.82 |  | 15.23 |  | **0.2016** |  | 13.65 |  | 15.61 |  | **0.4116** |
|  | Walking independently (months) | 36.97 |  | 33.70 |  | **0.1449** |  | 42.48 |  | 34.84 |  | **0.1408** |
|  | First words (months) | 24.50 |  | 30.17 |  | **0.3171** |  | 25.43 |  | 31.00 |  | **0.4121** |
|  | First smile (months) | 5.12 |  | 2.49 |  | **0.1456** |  | 6.74 |  | 2.48 |  | **0.0700** |
|  | Sitting at 9m | 13/44 | 29.55% | 15/65 | 23.08% | **0.1318** |  | 7/30 | 23.33% | 12/55 | 21.82% | **0.2099** |
|  | Sitting at 12m | 28/44 | 63.64% | 37/65 | 56.92% | **0.1244** |  | 15/30 | 50.00% | 29/55 | 52.73% | **0.1743** |
|  | Sitting at 18m | 35/44 | 79.55% | 52/63 | 84.13% | **0.1825** |  | 22/30 | 73.33% | 44/53 | 83.02% | **0.1273** |
|  | Walking at 18m | 10/51 | 19.61% | 7/73 | 9.59% | **0.0603** |  | 2/37 | 5.41% | 4/62 | 6.45% | **0.3316** |
|  | Walking at 24m | 13/46 | 28.26% | 25/67 | 37.31% | **0.0990** |  | 4/33 | 12.12% | 21/59 | 35.59% | **0.0097** |
|  | Walking at 36m | 22/44 | 50.00% | 37/61 | 60.66% | **0.0883** |  | 12/31 | 38.71% | 32/54 | 59.26% | **0.0348** |
|  | Walking at 60m | 33/43 | 76.74% | 46/56 | 82.14% | **0.1592** |  | 21/30 | 70.00% | 41/49 | 83.67% | **0.0807** |
|  | Speaking at 12m | 6/55 | 10.91% | 5/74 | 6.76% | **0.1755** |  | 3/42 | 7.14% | 2/63 | 3.17% | **0.2322** |
|  | Speaking at 18m | 9/52 | 17.31% | 10/71 | 14.08% | **0.1752** |  | 5/39 | 12.82% | 7/60 | 11.67% | **0.2406** |
|  | Speaking at 3y | 10/42 | 23.81% | 22/64 | 34.38% | **0.0898** |  | 5/30 | 16.67% | 17/53 | 32.08% | **0.0666** |
|  | Delayed sitting (after 9 months) | 31/44 | 70.45% | 50/65 | 76.92% | **0.1318** |  | 23/30 | 76.67% | 43/55 | 78.18% | **0.2099** |
|  | Delayed walking (after 18 months) | 41/51 | 80.39% | 66/73 | 90.41% | **0.0603** |  | 35/37 | 94.59% | 58/62 | 93.55% | **0.3316** |
|  | Delayed first smile (after 8 weeks) | 14/32 | 43.75% | 16/42 | 38.10% | **0.1673** |  | 12/22 | 54.55% | 14/36 | 38.89% | **0.1108** |
|  | Delayed speech | 60/63 | 95.24% | 88/89 | 98.88% | **0.1654** |  | 49/49 | 100.00% | 77/77 | 100.00% | **1.0000** |
|  | Ongoing speech/language problem | 40/46 | 86.96% | 67/70 | 95.71% | **0.0673** |  | 33/34 | 97.06% | 58/59 | 98.31% | **0.4689** |
|  | Non-verbal (over 3 years old) | 26/46 | 56.52% | 37/72 | 51.39% | **0.1297** |  | 22/33 | 66.67% | 31/61 | 50.82% | **0.0592** |
|  | ID | 62/65 | 95.38% | 76/82 | 92.68% | **0.2223** |  | 50/50 | 100.00% | 68/72 | 94.44% | **0.1171** |
|  | ID score (0=absent to 5=profound) | 2.22 |  | 1.92 |  | **0.1615** |  | 2.62 |  | 1.93 |  | **0.0031** |
|  | GDD score (0=absent to 4=severe) | 2.29 |  | 2.11 |  | **0.2005** |  | 2.58 |  | 2.13 |  | **0.0183** |
| **SOCIAL AND BEHAVIOURAL** | Autistic features | 47/67 | 70.15% | 66/91 | 72.53% | **0.1337** |  | 38/50 | 76.00% | 55/77 | 71.43% | **0.1400** |
|  | ASD diagnosis | 19/67 | 23.88% | 38/90 | 42.22% | **0.0274** |  | 15/50 | 30.00% | 33/76 | 43.42% | **0.0479** |
|  | Attention deficit/hyperactivity | 13/67 | 19.40% | 14/88 | 15.91% | **0.1431** |  | 7/50 | 14.00% | 13/75 | 17.33% | **0.1767** |
|  | Aggression | 19/67 | 28.36% | 31/90 | 34.44% | **0.1001** |  | 14/50 | 28.00% | 28/76 | 36.84% | **0.0917** |
|  | Challenging behaviour | 33/56 | 58.93% | 48/66 | 72.73% | **0.0426** |  | 25/43 | 58.14% | 43/58 | 74.14% | **0.0414** |
| **NEUROLOGICAL** | Hypotonia | 60/77 | 77.92% | 83/103 | 80.58% | **0.1336** |  | 52/60 | 86.67% | 75/88 | 85.23% | **0.1850** |
|  | Hypertonia | 8/77 | 10.39% | 4/103 | 3.88% | **0.0560** |  | 7/60 | 11.67% | 3/88 | 3.41% | **0.0416** |
|  | Contractures | 11/79 | 13.92% | 11/103 | 10.68% | **0.1443** |  | 11/62 | 17.74% | 10/88 | 11.36% | **0.1021** |
|  | Seizure reported | 21/72 | 29.17% | 27/98 | 27.55% | **0.1329** |  | 19/54 | 35.19% | 25/84 | 29.76% | **0.1182** |
|  | Ongoing seizures/epilepsy | 7/70 | 10.00% | 15/98 | 15.31% | **0.1145** |  | 7/52 | 13.46% | 13/83 | 15.66% | **0.1872** |
|  | Structural brain abnormalities | 23/47 | 48.94% | 27/67 | 40.30% | **0.1004** |  | 22/39 | 56.41% | 24/56 | 42.86% | **0.0720** |
| **VISION AND HEARING** | Strabismus | 26/63 | 41.27% | 36/77 | 46.75% | **0.1103** |  | 20/48 | 41.67% | 30/65 | 46.15% | **0.1361** |
|  | Other vision problem | 22/62 | 35.48% | 23/77 | 29.87% | **0.1129** |  | 16/48 | 33.33% | 17/65 | 26.15% | **0.1173** |
|  | Hearing problem | 8/64 | 12.50% | 6/77 | 7.79% | **0.1452** |  | 7/49 | 14.29% | 5/65 | 7.69% | **0.1285** |
| **SLEEP AND RESPIRATORY** | Apnoea | 19/78 | 24.36% | 10/104 | 9.62% | **0.0047** |  | 16/60 | 26.67% | 9/89 | 10.11% | **0.0058** |
|  | Sleep disturbance | 31/78 | 39.74% | 34/104 | 32.69% | **0.0767** |  | 22/60 | 36.67% | 27/89 | 30.34% | **0.1016** |
|  | ENT problem | 23/78 | 29.49% | 24/104 | 23.08% | **0.0841** |  | 17/60 | 28.33% | 22/89 | 24.72% | **0.1326** |
|  | Hyperventilation | 5/78 | 6.41% | 0/104 | 0.00% | **0.0134** |  | 5/60 | 8.33% | 0/89 | 0.00% | **0.0096** |
|  | Recurrent respiratory infections | 18/78 | 23.08% | 20/104 | 19.23% | **0.1191** |  | 13/60 | 21.67% | 12/89 | 13.48% | **0.0752** |
|  | Respiratory problems | 38/78 | 48.72% | 24/104 | 23.08% | **0.0002** |  | 29/60 | 48.33% | 18/89 | 20.22% | **0.0002** |
| **FEEDING AND GASTROINTESTINAL** | Feeding problems | 54/78 | 69.23% | 71/101 | 70.30% | **0.1286** |  | 47/59 | 79.66% | 61/86 | 70.93% | **0.0780** |
|  | Continuing feeding problems | 30/71 | 42.25% | 34/86 | 39.53% | **0.1220** |  | 28/53 | 52.83% | 29/72 | 40.28% | **0.0553** |
|  | Reflux/vomiting | 34/76 | 44.74% | 37/100 | 37.00% | **0.0722** |  | 29/57 | 50.88% | 31/86 | 36.05% | **0.0297** |
|  | Constipation | 12/76 | 15.79% | 16/100 | 16.00% | **0.1644** |  | 10/57 | 17.54% | 14/86 | 16.28% | **0.1759** |
|  | Tongue tie | 6/76 | 7.89% | 12/101 | 11.88% | **0.1404** |  | 4/57 | 7.02% | 12/86 | 13.95% | **0.0988** |
|  | Feeding tube | 25/76 | 32.89% | 20/101 | 19.80% | **0.0200** |  | 24/57 | 42.11% | 20/86 | 23.26% | **0.0089** |
| **MUSCULOSKELETAL** | Hypermobility | 16/73 | 21.92% | 26/86 | 30.23% | **0.0720** |  | 9/55 | 16.36% | 23/73 | 31.51% | **0.0245** |
|  | Scoliosis | 10/73 | 13.70% | 10/86 | 11.63% | **0.1747** |  | 9/55 | 16.36% | 8/73 | 10.96% | **0.1390** |
|  | Pectus excavatum/carinatum | 6/73 | 8.22% | 9/86 | 10.47% | **0.1928** |  | 5/55 | 9.09% | 9/73 | 12.33% | **0.1942** |
|  | Craniosynostosis | 6/73 | 8.22% | 9/87 | 10.34% | **0.1949** |  | 5/55 | 9.09% | 8/74 | 10.81% | **0.2227** |
|  | Any musculoskeletal problem | 50/74 | 72.97% | 64/87 | 73.56% | **0.0976** |  | 37/56 | 66.07% | 55/74 | 74.32% | **0.0915** |
|  | Pes planus | 6/73 | 8.22% | 8/89 | 8.99% | **0.2179** |  | 4/56 | 7.14% | 7/74 | 9.46% | **0.2274** |
|  | Dysmorphic hands/feet | 35/74 | 47.30% | 46/89 | 51.69% | **0.1071** |  | 31/57 | 54.39% | 35/74 | 47.30% | **0.1016** |
|  | Any hand/foot problems | 41/73 | 56.16% | 51/89 | 57.30% | **0.1252** |  | 33/56 | 58.93% | 40/74 | 54.05% | **0.1218** |
|  | Dental abnormalities | 28/48 | 58.33% | 36/63 | 57.14% | **0.1525** |  | 22/33 | 66.67% | 34/52 | 65.38% | **0.1843** |

Supplementary Material 3 - Summary of the phenotypes observed in each patient group. Where measuring the presence of a particular feature, counts are given as a proportion of all patients that were assessed for that feature. Numerical data is presented as the mean of all those assessed for that feature in each group. p values below 0.05 are highlighted in red, with darker shading representing a greater degree of significance.
