# Supplementary material for: Broadening the inherited ASXL3 spectrum and unveiling molecular mechanisms through detailed genotypic-phenotypic analyses
Source: Genet Med Open. 2026 May 28;4:104409. doi: 10.1016/j.gimo.2026.104409 (PMC13393785; doi:10.1016/j.gimo.2026.104409)
Supplement: Supplemental Material 4 [file mmc4.docx]

Supplementary Material 4 – Summary of phenotypic features in recurrent variants

The c.1210C>T p.(Gln404*) pathogenic (PVS1, PM2, PS2_mod, PS4_mod) nonsense variant in exon 11 is predicted to result in NMD. It has been identified in five individuals (including two siblings with the same apparent *de novo* variant).

|  | **Dillon et al.^34^**  **(L131)** | **Bainbridge et al.^4^**  **(L72)** | **Svantnerova et al.^35^**  **(L104)** | **Woods et al.^5^**  **(U32)** | **Woods et al.^5^**  **(U33)** | **Total** |
| --- | --- | --- | --- | --- | --- | --- |
| **Age** | Unknown | 9 months | Unknown | 1.2 years | 1.2 years | Known mean age = 1.2 years |
| **Sex** | Unknown | Unknown | Unknown | M | F | ½ male  ½ female |
| **Neonatal problems** | Unknown | Y | Unknown | Y (prematurity, poor feeding, jaundice) | Y (prematurity, poor feeding, jaundice) | 3/3 |
| **Growth problems** | Unknown | Y | Unknown | Y | Y | 3/3 |
| **Developmental delay, clinical impression** | Global delay, severe | Global delay, severe | Speech impairment, unknown severity | Too young to assess | Too young to assess | 3/3 delay  2/2 severe or moderate-to-severe |
| **Independently mobile** | Unknown | N/A – deceased nine months | Unknown | N/A – too young to assess | N/A – too young to assess | N/A |
| **Non-verbal** | Unknown | N/A | Unknown | N/A – too young to assess | N/A – too young to assess | N/A |
| **Autistic traits/ diagnosis** | Unknown | N/A | Unknown | N/A – too young to assess | N/A – too young to assess | N/A |
| **Head imaging abnormal** | Unknown | Unknown | Unknown | Y (subtle signs ‘cramped’ foramen magnum, agenesis corpus callosum) | Y (subtle signs ‘cramped’ foramen magnum) | 2/2 |
| **Chronic feeding problems** | Unknown | Y | Y | Y | Y | 4/4 |
| **Ever had need for assisted feeding (e.g. NGT, PEG)** | Unknown | Y | Unknown | Y | Y | 3/3 |
| **Hypotonia** | Y | N (hypertonia) | N (hypertonia, spastic contractures) | Y | Y | 3/5 |
| **Epilepsy** | Unknown | N | Unknown | N | N | 0/3 |
| **Strabismus** | Unknown | N | Unknown | N | N | 0/3 |
| **Behavioural problems** | Y | N/A | Unknown | N | N | 1/3 |
| **Mental health issues** | Unknown | N/A | Unknown | N/A | N/A | N/A |
| **Special education** | Unknown | N/A | Unknown | N/A | N/A | N/A |
| **Sleep issues** | Y | N | Unknown | N | Y | 2/4 |
| **Apnoea/ breathing concerns** | Unknown | N | Unknown | Y | N | 1/3 |
| **Recurrent infections** | Unknown | N | Unknown | N | N | 0/3 |
| **Genito-renal abnormality** | Unknown | Y (bladder dysfunction) | Unknown | N | N | 1/3 |
| **High palate** | Unknown | Y | Unknown | Y | Y | 3/3 |
| **Teeth problems** | Unknown | N/A | Unknown | N/A | N/A | N/A |
| **Hypertrichosis** | Y | N | Unknown | N | N | 1/4 |

The c.3106C>T, p.(Arg1036*) pathogenic (PVS1_str, PM2, PS2_mod, PS4_mod) nonsense variant in exon 12 results in greater than 10% protein loss. It has been identified in eight unrelated individuals (including two siblings with the same apparent *de novo* variant).

|  | **Koboldt et al.^9^**  **(L76)** | **Koboldt et al.^9^**  **(L77)** | **Schirwani et al.^3^**  **(L34)** | **Kuechler et al.^14^**  **(L80)** | **Myers et al.^59^**  **(L90)** | **Woods et al.^5^**  **(U24)** | **Heide et al.^64^**  **L156** | **Duan et al.^65^**  **L157** | **Total** |
| --- | --- | --- | --- | --- | --- | --- | --- | --- | --- |
| **Age** | 16 years | 15 years | 16.2 years | 4 years | 6 years | 0.9 years | N/A-foetus (TOP) | 3 years | Mean age = 8.7 years |
| **Sex** | F | F | M | F | M | M | F | M | 4/8 male  4/8 female |
| **Neonatal problems** | N | N (prematurity 36 weeks) | N | Y | Unknown | Y | N/A | Unknown | 2/5 |
| **Growth problems** | N | Y (small for age) | N | Y (failure-to-thrive) | Unknown | Y (slow weight gain) | N/A | Unknown | 3/5 |
| **Overall developmental delay clinical impression** | Global delay, severe | Global delay, severe | Global delay, severe | Global delay, severe | Global delay, severe | N/A – too young to assess | N/A | Global delay, unknown severity | 6/6 delay  5/6 severe |
| **Independently mobile** | Unknown | Unknown | Y | N | Y | N/A | N/A | Unknown | 2/3 |
| **Non-verbal** | Y | Y | Y | Y | Y | N/A | N/A | Unknown | 5/5 |
| **Autistic traits/ diagnosis** | Y | Y | Y | Y | N | N/A | N/A | Unknown | 4/5 |
| **Head imaging abnormal** | Y (hypoplasia of corpus callosum with ventriculomegaly) | N | N | N | N | N | Y (short corpus callosum) | Unknown | 2/7 |
| **Chronic feeding problems** | Y | Y | N | Y | Unknown | Y | N/A | Unknown | 4/5 |
| **Ever had need for assisted feeding (e.g. NGT, PEG)** | Unknown | Unknown | N | N | Unknown | Y | N/A | Unknown | 1/3 |
| **Hypotonia** | Y | Y | Y | Y | Y | Y | N/A | Unknown | 6/6 |
| **Epilepsy** | Y | Y | N | N | Y | N | N/A | Unknown | 3/6 |
| **Strabismus** | Unknown | Unknown | Y | Y | Unknown | N | N/A | Unknown | 2/3 |
| **Behavioural problems** | Y | Y | Y | Unknown | Unknown | N | N/A | Unknown | 3/4 |
| **Mental health issues** | Unknown | Unknown | Unknown | Unknown | Unknown | N/A | N/A | Unknown | Unknown |
| **Special education** | Y | Y | Y | Unknown | Unknown | N/A | N/A | Unknown | 3/3 |
| **Sleep issues** | Unknown | Unknown | Unknown | Unknown | Unknown | N | N/A | Unknown | 0/1 |
| **Apnoea/ breathing concerns** | Unknown | Unknown | Unknown | Unknown | Unknown | N (laryngomalacia resolved) | N/A | Unknown | 0/1 |
| **Recurrent infections** | Unknown | Unknown | Unknown | Unknown | Unknown | N | N/A | Unknown | 0/1 |
| **Genito-renal abnormality** | Unknown | Unknown | Unknown | Unknown | Unknown | N | N/A | Unknown | 0/1 |
| **High palate** | Y | Y | Y | Y | Unknown | Y | N/A | Unknown | 5/5 |
| **Teeth problems** | Y (prominent incisors) | Y (prominent incisors) | Y (large incisors) | Unknown | Unknown | N/A | N/A | Unknown | 3/5 |
| **Hypertrichosis** | Y (hirsutism) | Y (hirsutism) | Unknown | N | Unknown | N | N/A | Unknown | 2/4 |

The c.3349C>T p.(Arg1117*) pathogenic (PVS1_str, PM2, PS2_mod, PS4_mod) variant in exon 12 is predicted to result in greater than 10% of protein loss. It has been identified in four unrelated individuals.

|  | **Cuddapah et al.^53^**  **L70** | **Hegde et al.^67^**  **L135** | **Zhang et al.^42^**  **L158** | **Woods et al.^5^**  **U10** | **Total** |
| --- | --- | --- | --- | --- | --- |
| **Age** | 13 years | Unknown | 2.75 years | 1.1 years | Known mean age = 5.6 years |
| **Sex** | F | Unknown | F | M | 1/3 male  2/3 female |
| **Neonatal problems** | Y (dusky episodes, laryngomalacia) | Unknown | Unknown | Y (feeding) | 2/2 |
| **Growth problems** | Y (failure-to-thrive) | Unknown | Y | Y (failure-to-thrive) | 3//3 |
| **Overall developmental delay clinical impression** | Global delay, severe | Global delay, profound | Global delay, unknown severity | Global delay, moderate | 4/4 delay  2/3 severe |
| **Independently mobile** | Y | Unknown | Unknown | N | ½ |
| **Non-verbal** | Y | Unknown | Unknown | Y | 2/2 |
| **Autistic traits/ diagnosis** | Unknown | Unknown | Unknown | N | 0/1 |
| **Head imaging abnormal** | Y (thin corpus callosum, absent rostrum, small frontal lobes) | Y (diencephalon abnormality) | Unknown | Y (white matter changes) | 3/3 |
| **Chronic feeding problems** | Y | Unknown | Y | Y | 3/3 |
| **Ever had need for assisted feeding (e.g. NGT, PEG)** | Y | Unknown | Unknown | Y | 2/2 |
| **Hypotonia** | Y | Y | Y | Y | 4/4 |
| **Epilepsy** | N (one seizure) | Y | Unknown | N | 1/3 |
| **Strabismus** | Unknown | Unknown | Unknown | Y | 1/1 |
| **Behavioural problems** | Unknown | Unknown | Unknown | N | 0/1 |
| **Mental health issues** | Unknown | Unknown | Unknown | N/A | - |
| **Special education** | Unknown | Unknown | Unknown | Y | 1/1 |
| **Sleep issues** | Unknown | Unknown | Unknown | Y | 1/1 |
| **Apnoea/ breathing concerns** | Unknown | Unknown | Unknown | N | 0/1 |
| **Recurrent infections** | Unknown | Unknown | Unknown | N | 0/1 |
| **Genito-renal abnormality** | Unknown | Unknown | Unknown | N | 0/1 |
| **High palate** | Y | Unknown | Unknown | Y | 2/2 |
| **Teeth problems** | Unknown | Unknown | Unknown | Y | 1/1 |
| **Hypertrichosis** | Unknown | Unknown | Unknown | N | 0/1 |

The c.4330C>T p.(Arg1444*) pathogenic (PVS1_str, PM2, PS2_mod, PS4_mod) nonsense variant in exon 12 results in greater than 10% protein loss. It has been identified in eight unrelated individuals.

|  | **Schirwani et al.^3^**  **(L36)** | **Schirwani et al.^3^**  **(L44)** | **Schirwani et al.^3^**  **Woods et al.^5^**  **(PP10)** | **Balasubramanian et al.^32^**  **(L46)** | **Srivastava et al.^2^**  **(L60)** | **Woods et al.^5^**  **(U2)** | **Woods et al.^5^**  **(N1/U29)** | **Fu et al.^76^**  **(L127)** | **Total** |
| --- | --- | --- | --- | --- | --- | --- | --- | --- | --- |
| **Age** | 13 years | 5 years | 13.3 years | 11 years | 4 years | 5 years | 1.7 years | 7 months | Mean age = 6.7 years |
| **Sex** | M | M | M | M | M | M | M | F | 7/8 male  1/8 female |
| **Neonatal problems** | Y (feeding) | N | N | Y (pneumothorax) | Y (feeding) | Y (concerns regarding physical features) | N | Unknown | 4/7 |
| **Growth problems** | N | N | Y (small for age) | N | Y (failure-to-thrive) | N | Y (small for age) | Unknown | 3/7 |
| **Developmental delay, clinical impression** | Speech delay, severe | Global delay, unknown severity | Global delay, severe | Global delay, mild-to-moderate | Global delay, severe | Global delay, moderate-to-severe | Global delay, moderate | Global delay, unknown severity | 7/7 delay  4/6 severe or moderate-to-severe |
| **Independently mobile** | Y | Unknown | N | Y | N | Y | N | Unknown | 3/6 |
| **Non-verbal** | N | Unknown | N | N | Y | Y | Y | Unknown | 3/6 |
| **Autistic traits/ diagnosis** | N | Y | Y | Y | Unknown | Y | N/A | Unknown | 4/5 |
| **Head imaging abnormal** | N | Unknown | N | Y (minor terminal myelination defects) | Y (cerebellar vermis hypoplasia, shortened corpus callosum) | Y (minimal non-specific under development and immaturity of white matter) | Y (brain compression from craniosynostosis) | Unknown | 4/6 |
| **Chronic feeding problems** | N | Unknown | Y | N | Y | N | N | Unknown | 2/6 |
| **Ever had need for assisted feeding (e.g. NGT, PEG)** | N | Unknown | Y | N | Y | N | N | Unknown | 2/6 |
| **Hypotonia** | Unknown | Y | Y | Y | Y | Y | Y | Y | 7/7 |
| **Epilepsy** | N | Unknown | N | N (febrile seizures only) | Unknown | N | N | Unknown | 0/5 |
| **Strabismus** | Unknown | Unknown | Y | N | N | N | N | Unknown | 1/5 |
| **Behavioural problems** | Y | Unknown | Y | Y | Y | Y | N | Unknown | 5/6 |
| **Mental health issues** | N | Unknown | Y | Y (Tourette’s) | Unknown | N | N/A | Unknown | 2/3 |
| **Special education** | Y | Unknown | Y | Y | Unknown | Y | N/A | Unknown | 4/4 |
| **Sleep issues** | Y | Unknown | Y | Unknown | Unknown | Y | Y | Unknown | 4/4 |
| **Apnoea/ breathing concerns** | Y | Unknown | N | Unknown | Unknown | N | Y | Unknown | 2/4 |
| **Recurrent infections** | Y | Unknown | N | Unknown | Y | N | N | Unknown | 2/5 |
| **Genito-renal abnormality** | Y (hypospadias) | Unknown | N | Unknown | Unknown | Y (duplex kidney) | Y (increased volume kidneys) | Unknown | 3/4 |
| **High palate** | Unknown | Unknown | Y | Y | Unknown | Y | Y | Unknown | 4/4 |
| **Teeth problems** | Unknown | Unknown | Y | Unknown | Unknown | Y | Y | Unknown | 3/3 |
| **Hypertrichosis** | N | Unknown | Y | Unknown | Unknown | N | N | Unknown | 1/4 |

The c.4399C>T p.(Arg1467*) likely pathogenic (PVS1_str, PS2_mod, PS4_mod) nonsense variant in exon 12 is predicted to result in greater than 10% protein loss. It has been seen in five unrelated individuals.

|  | **Schirwani et al.^3^**  **L26** | **Schirwani et al.^3^**  **L27** | **Schirwani et al.^3^**  **L36** | **Yu et al.^16^**  **L96** | **Woods et al.^5^**  **U15** | **Total** |
| --- | --- | --- | --- | --- | --- | --- |
| **Age** | 2.3 years | 9.9 years | 4 years | 26 years | 8.6 years | Mean age = 10.2 years |
| **Sex** | F | F | M | F | M | 2/5 male  3/5 female |
| **Neonatal problems** | N | Y (prematurity 35 weeks) | N | Y | N | 2/5 |
| **Growth problems** | Y (failure-to-thrive) | Unknown | Unknown | Y (obesity) | Y (failure-to-thrive) | 3/3 |
| **Overall developmental delay clinical impression** | Global delay, severe | Global delay, severe | Global delay, severe | Intellectual disability, moderate (assessed in adulthood) | Global delay, moderate | 5/5 delay/ ID  3/5 severe |
| **Independently mobile** | Y | Y | Unknown | Y | Y | 4/4 |
| **Non-verbal** | N | Y | N | N | N | 1/5 |
| **Autistic traits/ diagnosis** | Unknown | Y | Unknown | N | Y | 2/3 |
| **Head imaging abnormal** | Unknown | Y (dysplastic temporal horn, possible cortical dysplasia, unusual configuration brainstem and pons) | Unknown | Y (prominent sylvian fissures with extra axial CSF spaces) | N | 2/3 |
| **Chronic feeding problems** | Y | Y | Y | Y (in infancy) | Y | 5/5 |
| **Ever had need for assisted feeding (e.g. NGT, PEG)** | N | Y | Unknown | Unknown | N | 1/3 |
| **Hypotonia** | Y | Y | Unknown | Y | Y | 4/4 |
| **Epilepsy** | N | N | Unknown | N | N | 0/4 |
| **Strabismus** | Unknown | Unknown | Unknown | Y | N | 1/2 |
| **Behavioural problems** | Unknown | Y | Unknown | Unknown | Y | 2/2 |
| **Mental health issues** | Unknown | Unknown | Unknown | Unknown | N | 0/1 |
| **Special education** | Unknown | Y | Unknown | Unknown | Y | 2/2 |
| **Sleep issues** | Unknown | Unknown | Unknown | Unknown | Y | 1/1 |
| **Apnoea/ breathing concerns** | Unknown | Unknown | Unknown | Unknown | N | 0/1 |
| **Recurrent infections** | Unknown | Unknown | Unknown | Unknown | Y | 1/1 |
| **Genito-renal abnormality** | Unknown | Unknown | Unknown | Unknown | N | 0/1 |
| **High palate** | Y | Unknown | Y | Unknown | N | 2/3 |
| **Teeth problems** | Unknown | Unknown | Unknown | Unknown | Y (caries, extractions) | 1/1 |
| **Hypertrichosis** | Unknown | Unknown | Unknown | Unknown | Y | 1/1 |
